# Evolutionary coupling analysis identifies the impact of disease-associated variants at less-conserved sites

**DOI:** 10.1093/nar/gkz536

**Published:** 2019-06-14

**Authors:** Donghyo Kim, Seong Kyu Han, Kwanghwan Lee, Inhae Kim, JungHo Kong, Sanguk Kim

**Affiliations:** Department of Life Sciences, Pohang University of Science and Technology, Pohang 790-784, Korea

## Abstract

Genome-wide association studies have discovered a large number of genetic variants in human patients with the disease. Thus, predicting the impact of these variants is important for sorting disease-associated variants (DVs) from neutral variants. Current methods to predict the mutational impacts depend on evolutionary conservation at the mutation site, which is determined using homologous sequences and based on the assumption that variants at well-conserved sites have high impacts. However, many DVs at less-conserved but functionally important sites cannot be predicted by the current methods. Here, we present a method to find DVs at less-conserved sites by predicting the mutational impacts using evolutionary coupling analysis. Functionally important and evolutionarily coupled sites often have compensatory variants on cooperative sites to avoid loss of function. We found that our method identified known intolerant variants in a diverse group of proteins. Furthermore, at less-conserved sites, we identified DVs that were not identified using conservation-based methods. These newly identified DVs were frequently found at protein interaction interfaces, where species-specific mutations often alter interaction specificity. This work presents a means to identify less-conserved DVs and provides insight into the relationship between evolutionarily coupled sites and human DVs.

## INTRODUCTION

As sequencing technology has advanced, many non-synonymous variants have been identified in a number of genome-wide association studies (GWASs). Therefore, it is important to evaluate the impacts of these variants on human health and disease, because functional consequences may vary among variants. To this end, many computational methods have been developed to predict the impacts of genetic variants (1–5). Most methods depend on evolutionary conservation by assuming that functionally important residues in proteins are conserved and that variants in well-conserved residues have a greater impact. These conservation-based methods are mostly used to sort between disease-associated and neutral variants in GWASs (6,7).

However, conservation-based methods cannot predict the impact of disease-associated variants (DVs) for less-conserved residues, though DVs have been found in residues that are less conserved (8). For instance, the K329E variant of acyl-CoA dehydrogenase (ACADM) is associated with medium-chain acyl-CoA dehydrogenase (MCAD) deficiency, which is characterized by hypoglycemia and sudden death (MIM: 201450) (9,10). However, this variant could not be identified by conservation-based methods, such as SIFT and PROVEAN (11,12), because it occurs at a site which is less conserved in multiple sequence alignments (MSAs). Thus, another evolutionary approach is needed to identify variants of residues that are not well conserved but are associated with human disease.

Coevolutionary analyses have emerged as the principle for predicting DVs. Evolutionarily coupled residues are determined on the basis of the statistical power of covariation patterns in the MSA. Covariation often occurs when a variant at a functionally important residue results in the development of compensatory variants at cooperative residues to avoid a loss of function. Thus, evolutionary coupling suggests that the two residues are linked to carry out important structural or functional roles. Specifically, the quantification of covariation strength among all possible residues predicts variant impacts on proteins (13–17). Moreover, the analysis of pairwise and direct covariation strength among residues, excluding the effect from other positions (i.e. direct coupling analysis), has been applied to predict three-dimensional contacts in protein and RNA structures (18–25).

Here, we hypothesized that our approach based on evolutionary coupling ‘number’, which focuses on how many evolutionary couplings a residue has, would optimize the discovery of DVs at less-conserved sites. Functional properties, especially for less-conserved residues, such as protein conformational change (26), allosteric regulation (27), substrate specificity determining (28) and protein–protein interactions (PPIs) (29), were known to be associated with evolutionary coupling number. Moreover, the residues with a high coupling number were differentially located from the residues with high coupling strength, although they share the coevolution principle. Specifically, the high coupling numbers of those residues were driven by multiple moderate covariation scores rather than a few strong scores (30).

**Figure 1.**
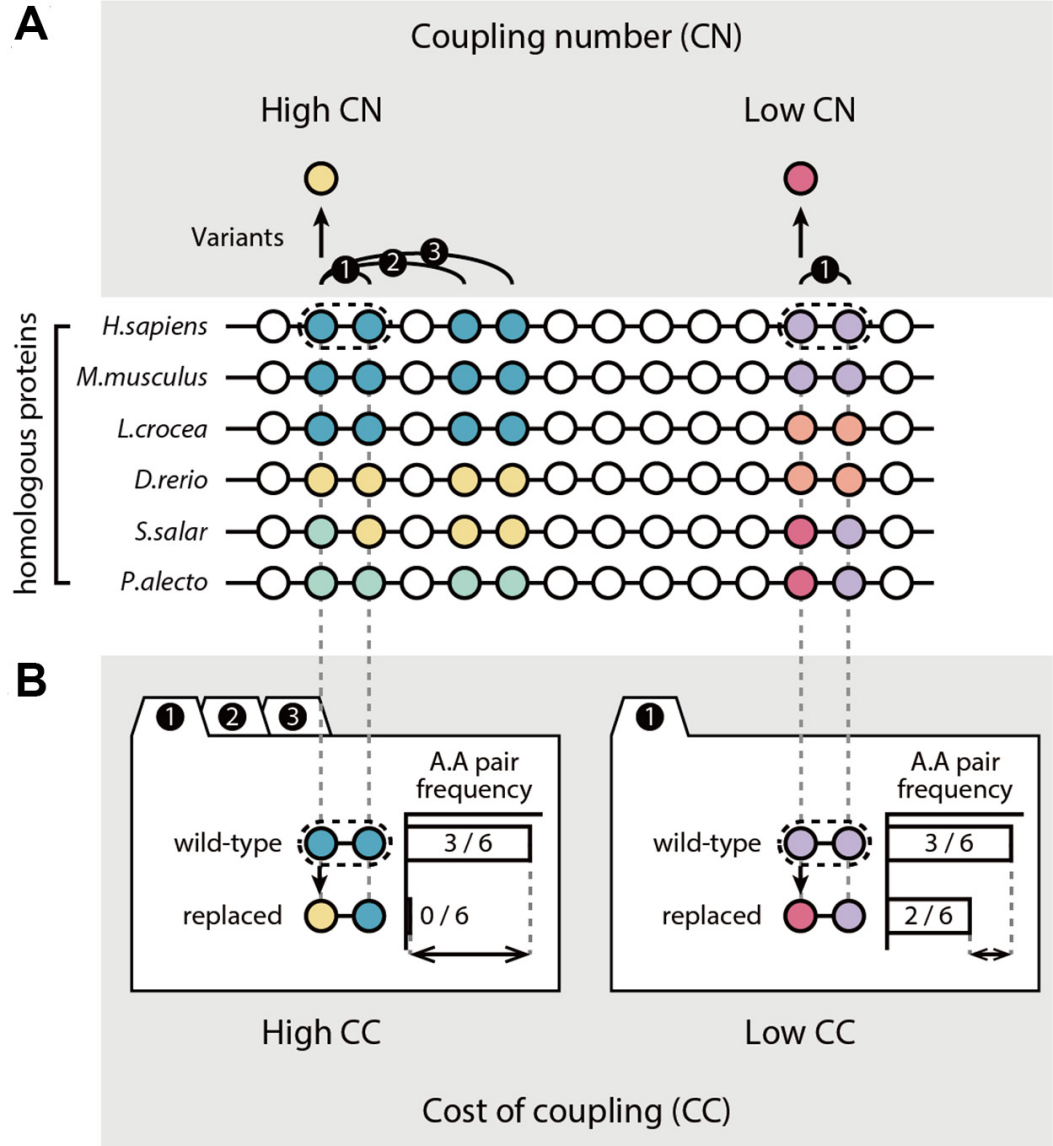
Outline of variant impact prediction using coevolution (CE) scores. The CE scores for amino acid variants were calculated by multiplying two coevolutionary matrices: the CN and the CC. (**A**) Schematic diagram of the CN calculation. The colors of the circles indicate the types of amino acids. Curved lines represent evolutionary couplings between sites, and the numbers above the lines denote the order of the couplings at a site. Circles and straight lines represent the MSAs for the homologous proteins. (**B**) Schematic diagram of CC calculation. CC scores compared the frequency of amino acid pairs between wild-type and variant pairs at coupled sites from MSAs.

In this study, we used the evolutionary coupling number to develop a computational method to predict the impacts of DVs at less-conserved sites. Specifically, we predicted variant impacts by developing the coevolution (CE) score, which is calculated by multiplying two coevolutionary matrices: the coupling number (CN) and the cost of coupling (CC) (Figure 1). The CN indicates the evolutionary importance of the residue where a variant occurs by measuring how many residues are evolutionarily coupled with the residue at the variant site. When a residue with a variant is highly coupled, the CN becomes high and indicates functional importance for the residue. In contrast, the CC measures the influence of the amino acid change of the variant on evolutionary couplings and indicates the evolutionary tolerance of the altered amino acid pairs in the coupled residues. Specifically, when altered pairs have a low frequency between homologous proteins relative to wild-type pairs, CC becomes high and indicates that the variants are intolerant.

We validated the ability of the CE score to accurately predict the impact of variants by experimentally measuring variant impacts from five saturated mutagenesis studies. We found that the CE score identified intolerant variants at sites that were less conserved and would not be identified by conventional methods. Moreover, integrating the CE score with conservation-based methods improved the ability to predict mutational impacts. When we applied CE scores to identify DVs in humans, we found that our method discovered 1261 disease-associated variants at less-conserved sites that could not be identified by current methods. Interestingly, DVs found by our method often occurred at the interfaces of PPIs on protein surfaces. In summary, the CE score enables the identification of functionally important DVs from GWASs. We also provide precalculated CE scores for all possible human gene variants on a user-friendly companion site (https://sbi.postech.ac.kr/w/CE).

## MATERIALS AND METHODS

### Generation of multiple sequence alignments (MSAs)

An average of 182 homologous sequences per protein were obtained from the UniRef90 database of nonredundant protein sequences (released 08/2017) (31) using PSI-BLAST (*E*-value < 0.001) (32). We omitted columns with a gap of more than 20% and with completely conserved regions and excluded proteins with fewer than 10 homologous sequences from the analysis. We aligned sequences that had <90% identity and were 0.7–1.3 × the length of the query sequence, using MUSCLE with the default options (33). The resulting MSAs of the human proteins are available from our companion site (https://sbi.postech.ac.kr/w/CE).

### Calculating coupling number (CN)

Evolutionary couplings of protein residues were chosen according to a length-dependent threshold based on the covarying strengths of residue pairs (13,14). We calculated the covarying strengths using the McLachlan-based substitution correlation method, which measures the correlations of substitution patterns between two different sites in an MSA using similarity scores based on a position-specific matrix (13,34–36). The length-dependent threshold was chosen by multiplying the protein lengths (L). Specifically, we determined a threshold to be twice the protein length (2L), which has been systematically examined to optimize the coupling analysis (26,27). In other words, in a protein with length L, there are 2L number of couplings. We counted and normalized the number of coupled pairs for each residue and defined that number as the CN. The normalization was assigned by converting CNs into the corresponding percentile rank scores that ranged from 0 to 1 to correct for the different score distributions among the proteins. The calculated CNs of variants in human proteins are also available at our companion site.

### Calculating cost of coupling (CC)

To examine how evolutionarily coupled sites are influenced by altering the amino acid type, we measured the evolutionary tolerance of altering amino acid pairs for the coupled residues. We assumed that evolutionarily unfavorable amino acid pairs, which are rarely observed in homologous proteins, have detrimental impacts. Specifically, we use the entropy of the amino acid pair distribution in two aligned columns that are evolutionarily coupled. The entropy of two evolutionarily coupled residues *i* and *j* is as follow:

$$\begin{equation*}{S_{i,j}} = \ ln\frac{{N!}}{{\mathop \prod \nolimits_{\alpha ,\beta } {n_{i,j}}\left( {\alpha ,\beta } \right)!}}\end{equation*}$$

*N* is the total number of amino acid pairs in the aligned columns of the residues *i* and *j*. *α* and *β* are wild-type amino acids on the residues *i* and *j*. ${n_{i,\ j}}( {\alpha ,\beta } )$ is the number of *α*-*β* amino acid pairs in aligned columns *i* and *j*. CC was measured by averaging entropy differences caused by a variant in residue *i* paired with its coupled residues. When a variant from *α* to *γ* occurs in the residue *i*, the CC of the variant is as follows:

$$\begin{equation*}\Delta {S_{i,\ j}}\ \left( {\alpha \to \gamma } \right) = \ - ln\frac{{{n_{i,\ j}}\left( {\gamma ,\beta } \right) + 1}}{{{n_{i,\ j}}\left( {\alpha ,\ \beta } \right)}}\end{equation*}$$

$$\begin{equation*}C{C_i}\ \left( {\alpha \to \gamma } \right) = \ \frac{{\mathop \sum \nolimits_{j \in C} \Delta {S_{i,j}}\left( {\alpha \to \gamma } \right)}}{{\left| C \right|}}\end{equation*}$$

*γ* is altered amino acid of residue *i*, and *β* is the wild-type amino acid for the residue *j*. *C* is a group of evolutionarily coupled partners of the residue *i*. The calculated CCs of all possible variants in human proteins are also available at our companion site.

### Calculating coevolution (CE) scores

The CE score was calculated by multiplying the CN and the CC. The formulation of CE scores is outlined in Figure 1 in more detail.

### Data set collection

We collected five proteins with impacts of variants, which were measured from saturation mutagenesis experiments (Table 1). For reliable and unbiased validation, we selected experiments in which more than 95% of all residues were replaced by more than five amino acids on average. We classified the variants as intolerant or tolerant according to changes in the protein activities of variants relative to that of the wild-type protein. LacI and lysozyme mutagenesis experiments provided the impacts of variants as the Boolean values according to the change in protein function or organism fitness: intolerant or tolerant. In contrast, TP53, APH(3′)-II and BLAT mutagenesis studies provided impacts as continuous values. In those experiments, we defined intolerant variants as those that had <50% of wild-type activity.

**Table 1.** Collection of five high-throughput saturated mutagenesis studies used for analysis

Identifier	Number of variants	Sequence length	Number of variants per residue	Ratio of the residues with variants	PMID
LacI	4041	329	12.28	1.00	8046748
Lysozyme	2015	164	12.29	0.99	1942069
TP53	2314	393	5.89	1.00	27328919
APH(3′)-II	4961	264	18.79	1.00	24914046
BLAT	5198	286	18.17	1.00	24567513

The number of variants per site was calculated by dividing the number of variants by sequence length.

To assess the ability of the CE score to discriminate between known disease-associated variants (DVs) and common polymorphic variants (CVs), we obtained DVs and CVs from the UniProt humsavar list (released 04/2018) (37), ClinVar (38) and ExAC (39). We collected 29 288 DVs annotated with ‘disease’ in the humsavar and 17 523 DVs annotated with ‘pathogenic’ in the ClinVar. Between the two DV sets, 10 051 DVs overlapped. We collected 39 167 CVs that are annotated with ‘polymorphism’ (variants with no known disease association) in the humsavar list. From the ExAC, 4244 CVs with high allele frequency (AF) were selected as CVs (AF > 0.1). Between the two CV sets, 2671 CVs overlapped.

### Conventional methods to predict mutational impacts of variants

To compare the CE score with scoring by conventional methods, we collected SIFT (5), Polyphen2 (1), PROVEAN (12), EVmutation (17) and CS (ConServation) scores (40). The SIFT scores were calculated using local installation of SIFT. The Polyphen2, PROVEAN and EVmutation scores of DVs and CVs were obtained using precalculated scores. The CS score is one of the conservation-based methods for predicting variant impacts by measuring entropy differences for the amino acid distribution in a single-aligned column with a variant (40).

### Prediction performance test

To evaluate prediction performances of the CE, CS, SIFT and integrated scores without specific thresholds, we computed receiver operating characteristics (ROC) curves. We defined true positive (TP) as the number of correctly predicted intolerant variants or DVs, true negative (TN) as the number of correctly predicted tolerant variants or CVs, false positive (FP) as the number of erroneously predicted tolerant variants or CVs and false negative (FN) as the number of erroneously predicted intolerant variants or DVs. The ROC curve was created by plotting the true positive rate, which is the fraction of the TP over TP + FP, and the false positive rate, which is the fraction of FP over TN + FP, at various threshold settings. To measure prediction performance, we computed the area under the ROC curve (AUC).

To evaluate the predictive abilities of the CE, CS, SIFT and integrated scores at specific thresholds, we determined the best threshold for each score using training sets by choosing a threshold that maximized accuracy. To ensure a fair comparison, we also determined thresholds for the SIFT score. The accuracy was calculated as follows:

$$\begin{equation*}{\rm{Accuracy\ }} = \frac{{TP + TN}}{{TP + FN + TN + FP}}\end{equation*}$$

Then, we employed several parameters to measure performance, including sensitivity, precision, accuracy, balanced accuracy, the Matthews correlation coefficient (MCC) and the F1 score using test sets. These performance parameters were calculated as follows:

$$\begin{equation*}{\rm{Sensitivity\ }} = \frac{{TP}}{{TP + FN}}\end{equation*}$$

$$\begin{equation*}{\rm{Precision\ }} = \frac{{TP}}{{TP + FP}}\end{equation*}$$

$$\begin{equation*}{\rm{Balanced\ accuracy\ }} = {\rm{\ }}\frac{{\left( {\frac{{TP}}{{TP + FN}} + \frac{{TN}}{{TN + FP}}} \right)}}{2}\end{equation*}$$

$$\begin{equation*}{\rm{MCC\ }} = \frac{{TP \times TN - FP \times FN}}{{\sqrt {\left( {TP + FP} \right)\left( {TP + FN} \right)\left( {TN + FP} \right)\left( {TN + FN} \right)} }}\ \end{equation*}$$

$$\begin{equation*}{\rm{F}}1{\rm{\ score\ }} = {\rm{\ }}2{\rm{\ }} \times \frac{{{\rm {\rm Precision }}\times {\rm Sensitivity}}}{{{\rm Precision }+ {\rm Sensitivity}}}\end{equation*}$$

The prediction performances of the scores were compared by random forest classifiers that construct multiple training sets and by fitting a decision tree (41). The Python package ‘sklearn.ensemble’ was applied and used to construct 1000 trees with maximum depth three. For valid measurements of prediction performances, we performed Monte Carlo cross-validation (100 times), randomly splitting the dataset into training (90%) and testing (10%) sets. The classifiers were constructed with a single feature (CE, CS or SIFT score) or two features (CE and CS scores or CE and SIFT scores) of the training set. To analyze the prediction performance of each classifier, sensitivity, precision, balanced accuracy, the MCC and the F1 score of prediction results of the testing set were measured.

### Principal component analysis (PCA)

For the Principal component analysis (PCA) of DVs and CVs, we used the standard R function ‘prcomp.’ The PCA was performed using eight scores (CN, CC, CE, CS, SIFT, Polyphen2, PROVEAN and EVmutation scores). To use the same directionality of each score as a measure of variant impact (with a high score indicating a larger impact), we multiplied −1 by SIFT, PROVEAN and EVmutation scores. We scaled each score to have unit variance before the analysis, using the scale = T option. We chose the first two principal components that cumulatively accounted for >88% of the total data variance.

### Analysis of structural features

To classify residues with DVs or CVs as interface or noninterface residues, we used Interactome INSIDER and Inferred Biomolecular Interaction Server (IBIS) databases (42,43). To ensure the quality of the data, we only used interfaces that had the highest confidences, which included interface residues calculated from PDB structures. Additionally, we classified the remaining residues as either ‘the rest of surface’ or the ‘protein core.’ To assign these classifications, we analyzed the PDB structures using NACCESS (44). We mapped human proteins onto PDB structures using UniProt entries in PDB, which are provided by RCSB PDB (https://www.rcsb.org/pdb/secondary.do?p=v2/secondary/other_download.jsp). We classified residues as ‘the rest of surface’ if they were in an accessible solvent area of the protein that was at least 10% of the residue's total surface. All other residues were classified as protein core. We only analyzed proteins with both INSIDER and PDB data, excluding 83 variants that were annotated as interface and protein core. Overall, we analyzed the structural features of 8191 variants for 567 proteins.

## RESULTS

### Prediction of mutational impacts using evolutionary coupling analysis

We created a computational method to predict the impacts of sequence variants at less-conserved sites based on evolutionary coupling analysis. The CE score was calculated by multiplying the CN and CC.

CN indicates the evolutionary importance of the residue with a variant and is measured by evolutionary coupling analysis (Figure 1A). To calculate the CN, we examined the evolutionary couplings between residues using homologous proteins. The CN is the normalized number of coupled residues that a variant has. We used evolutionary coupling to predict the impacts of variants at less-conserved residues, because it is reported that residues that are evolutionarily coupled with many other sites in the protein are important for allosteric regulation and protein conformational changes, even though they are less conserved between homologous protein sequences (26,27). A CN of 1 for a variant indicates that the variant occurs at the most coupled residue in the protein sequence, whereas a CN of 0 indicates that the variant occurs at the least coupled residue.

By contrast, CC measures the influence of an amino acid change on the evolutionarily coupled residues (Figure 1B). When a functionally important residue is replaced by a different amino acid, the impact depends on the type of amino acid that was replaced (45). To quantify the impact of the replacement, we compared the frequencies of the changes between the wild-type and variant amino acid pairs in the coupled residues of homologous proteins using MSAs. Amino acid pairs with high impact are usually less frequent, whereas those with low impact are usually more frequent (46). We determined the CC by estimating the entropy differences of the pair distributions for the coupled residues of wild-type and variant pairs. To examine variant impacts on evolutionarily coupled sites, we measured the average of entropy differences of the amino acid pairs at coupled sites.

### Validation of the CE score for predicting variant impacts

To confirm that the CN, CC and CE scores can predict the impact of variants, we analyzed the scores of amino acid variants with experimentally measured impacts. To this end, we used five high-throughput saturated mutagenesis studies (Table 1) (47–51). To ensure an unbiased evaluation of the scores for possible variants on a protein, we only chose the studies in which >95% of the amino acid residues in a protein sequence were replaced by five or more amino acids on average. For example, the lysozyme mutagenesis study provided phenotypic outcomes for 163 of the 164 residues, with an average of 12.28 variants per residue (coverage 99.39%). We classified variants as intolerant or tolerant according to experimental measurements. Intolerant variants had less protein activity or organismal fitness than tolerant ones.

**Figure 2.**
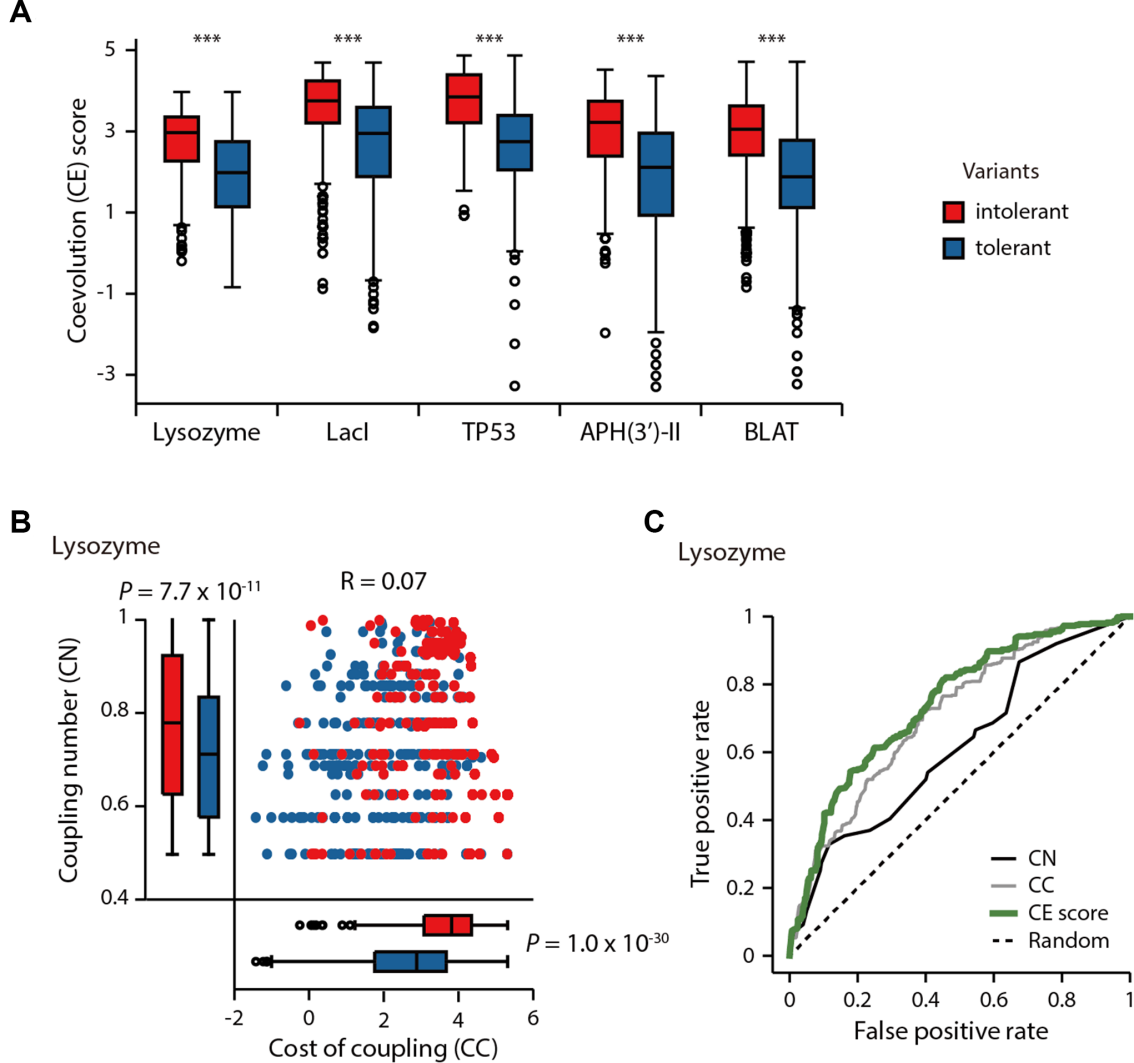
Systematic and unbiased performance test for variant impact predictions using five saturated mutagenesis studies. (**A**) CE scores of intolerant (red) and tolerant (blue) variants. Asterisks denote significant differences for CE scores (Mann–Whitney U test, ***P < 1.0 × 10^−39^). (**B**) CN and CC distributions of intolerant and tolerant lysozyme variants. (**C**) The ROC curves of the CN, CC and CE scores for predicting the impacts of variants from the lysozyme mutagenesis study (AUCs for CE, CN and CC scores were 0.74, 0.62 and 0.71, respectively).

We found that CN, CC and CE scores could be used to predict the impacts of the variants. Specifically, we calculated the CN, CC and CE scores for the variants (Supplementary Data S1). The CN, CC and CE scores of intolerant variants were significantly higher than those of tolerant variants, for all the experimental data sets (Figure 2A and 2B; Supplementary Figure S1). The average CN, CC and CE scores of the intolerant variants for the lysozyme data were 0.77, 3.61 and 2.75, respectively, whereas those of the tolerant variants were 0.71, 2.65 and 1.89, respectively (Mann–Whitney U test: CN, *P* = 7.7 × 10^−11^; CC, *P* = 1.0 × 10^−30^; CE, *P* = 1.7 × 10^−40^). For example, D10Y of lysozyme had an inhibitory effect on the plaque formation of bacteriophage and is predicted as an intolerant variant with a high CE score of 3.21. In contrast, K13F of lysozyme did not have the same effect on plaque sizes and is predicted as a tolerant variant with a low CE score of 1.32.

We also observed that integrating the CN and CC scores into the CE score improved the ability to predict the impacts of variants. To compare the predictive abilities of the scores, we analyzed the ROC curves. We found that the AUC for the CE score was higher than those of the CN and CC (Figure 2C and Supplementary Figure S2). The average AUCs for CN, CC and CE scores were 0.60, 0.72 and 0.75, respectively. We note that the CC and CN correlated poorly (Supplementary Figure S1, Pearson correlations ranged from −0.16 to 0.29), suggesting the distinct explanatory power of CN and CC for the impacts of variants. Therefore, the integration of CN and CC is necessary to accurately predict the variant impact.

### Integrating CE scores with conservation-based methods improved the prediction of variant impacts

**Figure 3.**
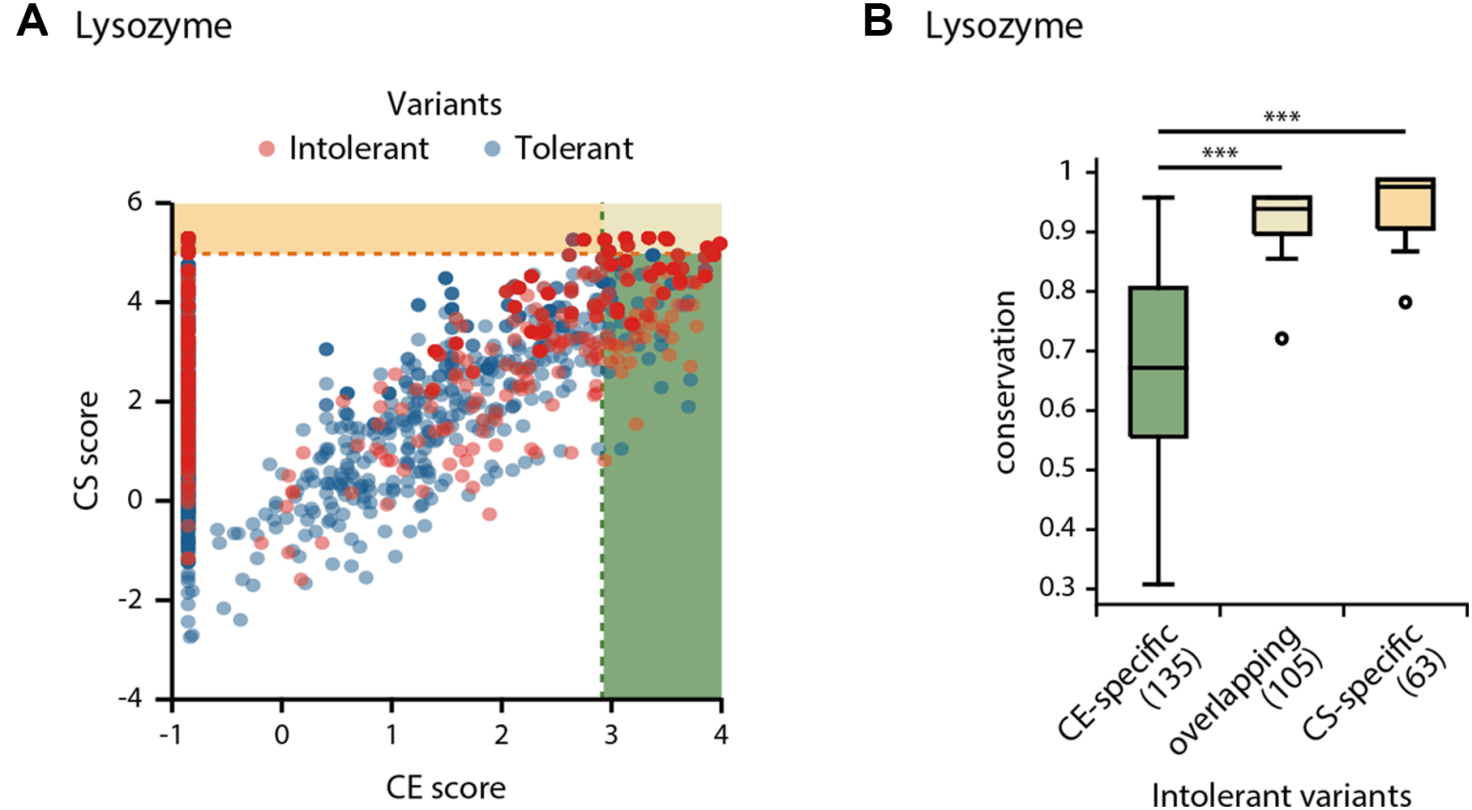
Prediction of the impacts of variants using CE and CS scores. (**A**) CE and CS scores of lysozyme intolerant (red) and tolerant variants (blue). The shaded regions represent where the CE (green) or CS (yellow) scores exceed the optimal thresholds. (**B**) Conservation of the CE-specific, overlapping or CS-specific intolerant variant sites.

We compared the predictions made by the CE scores with the two different conservation-based methods, the conservation (CS) and SIFT scores (4,5). We confirmed that the intolerant variants specifically identified by CE scores occurred at significantly less-conserved sites than variants identified by the CS or SIFT scores (Figure 3; Supplementary Figures S3 and 4; Mann–Whitney U test). For example, for lysozyme variants, the intolerant variants specific to the CE score were found in less-conserved residues (*n* = 135, average conservation = 0.67) compared to those predicted by both CE and CS scores (*n* = 105, average conservation = 0.92, *P* = 5.0 × 10^−34^) or only CS scores (*n* = 63, average conservation = 0.95, *P* = 2.0 × 10^−27^). In particular, the number of newly identified intolerant variants by the CE scores is higher than those by other conservation-based methods. For example, the CE score identified 135 of the 458 intolerant lysozyme variants that the CS score could not identify (Figure 3B; 29.48%); further, the SIFT score only identified 89 variants (19.4%).

Given that the CE score and the conservation-based scores targeted intolerant variants with different conservation levels, we anticipated that integrating both scores would improve the ability to predict variant impacts. To prove this, we used a Monte Carlo cross-validation and applied two different standalone classifiers that relied on either the CE or the conservation-based scores. Briefly, we searched the optimal thresholds of CE or the conservation-based scores of the training set to predict variant impacts. Then, we classified a variant as intolerant if at least one score of the variant exceeded the threshold. At last, we measured various matrices of prediction performance.

Consistent with our expectations, we found that integrating CE scores with conservation-based scores substantially improved the predictions of variant impacts (Supplementary Figure S5). Specifically, the F1 score, MCC and balanced accuracy of the integrated score were significantly higher than for those of the conservation-based scores. For example, the F1 score of the integrated score (CE+CS) for lysozyme variants was 0.56 ± 0.01 (mean value ± standard error computed over 100 cross-validations), which was significantly higher than the F1 score for the CS score (0.40 ± 0.01; *P* = 9.6 × 10^−39^, Student's *t*-test). We attribute the improved performance of the integrated approach to the correct identification of intolerant variants while limiting the number of erroneously identified tolerant variants. Specifically, we observed that the gain of sensitivity of the integrated approaches surpassed the loss of precision in all of the mutagenesis studies (Supplementary Figure S6). Here, we defined sensitivity and precision by the fraction of correctly identified intolerant variants among actual intolerant variants and among the variants predicted as intolerant, respectively. In the case of lysozyme variants, the sensitivity of the integrated score (CE+CS) improved to 0.56 ± 0.01, compared to the CS only score of 0.41 ± 0.01. When measuring precision, we found that the integrated score was comparable to the CS score (CE+CS, precision = 0.61 ± 0.01; CS, precision = 0.64 ± 0.01).

We considered the possibility that the intolerant variants identified using the integrated score could also be found by relaxing the threshold of the conservation-based scores; however, this was not the case. We found that the integrated score outperformed the scores obtained by adjusting the threshold of conservation-based scores, because using the integrated score includes more intolerant variants without taking a significant amount of tolerant variants. To validate this finding, we measured precision after changing the threshold for the conservation-based scores and comparing the resulting values with the integrated score. We found that lowering the threshold for the CS scores resulted in significantly less precision relative to the precision of the integrated scores (CE+CS) (Supplementary Figure S7; *P* = 2.2 × 10^−34^ to 9.4 × 10^−85^, Mann–Whitney U test).

To prove that integration of the CE score substantially improves the predictions of variant impacts, we also applied a machine learning method, random forest classifier. Specifically, we compared the prediction performances of the classifiers with a single feature (CE, CS or SIFT scores) and those with combining features (CE and CS or SIFT scores). The various matrices showed that the combination of the CE score with the conservation-based scores improved the prediction of variant impact (Supplementary Figure S8). For example, the F1 score of the classifier combining the CE and CS scores was an average of 0.69, which was much higher than that using only the CS score (0.63 on average). We also attribute the improved performance of the CE score to an increased sensitivity while keeping the precision (Supplementary Figure S9). Application of the random forest classifiers enables a determination of the significance of the CE scores in terms of the feature importance. The averages of the feature importance of the CE scores ranged from 0.32 to 0.49 (Supplementary Figure S10), which suggests that the CE score contributed to predicting the variant impacts in the classifiers.

### CE scores identify disease-associated variants at less-conserved sites

**Figure 4.**
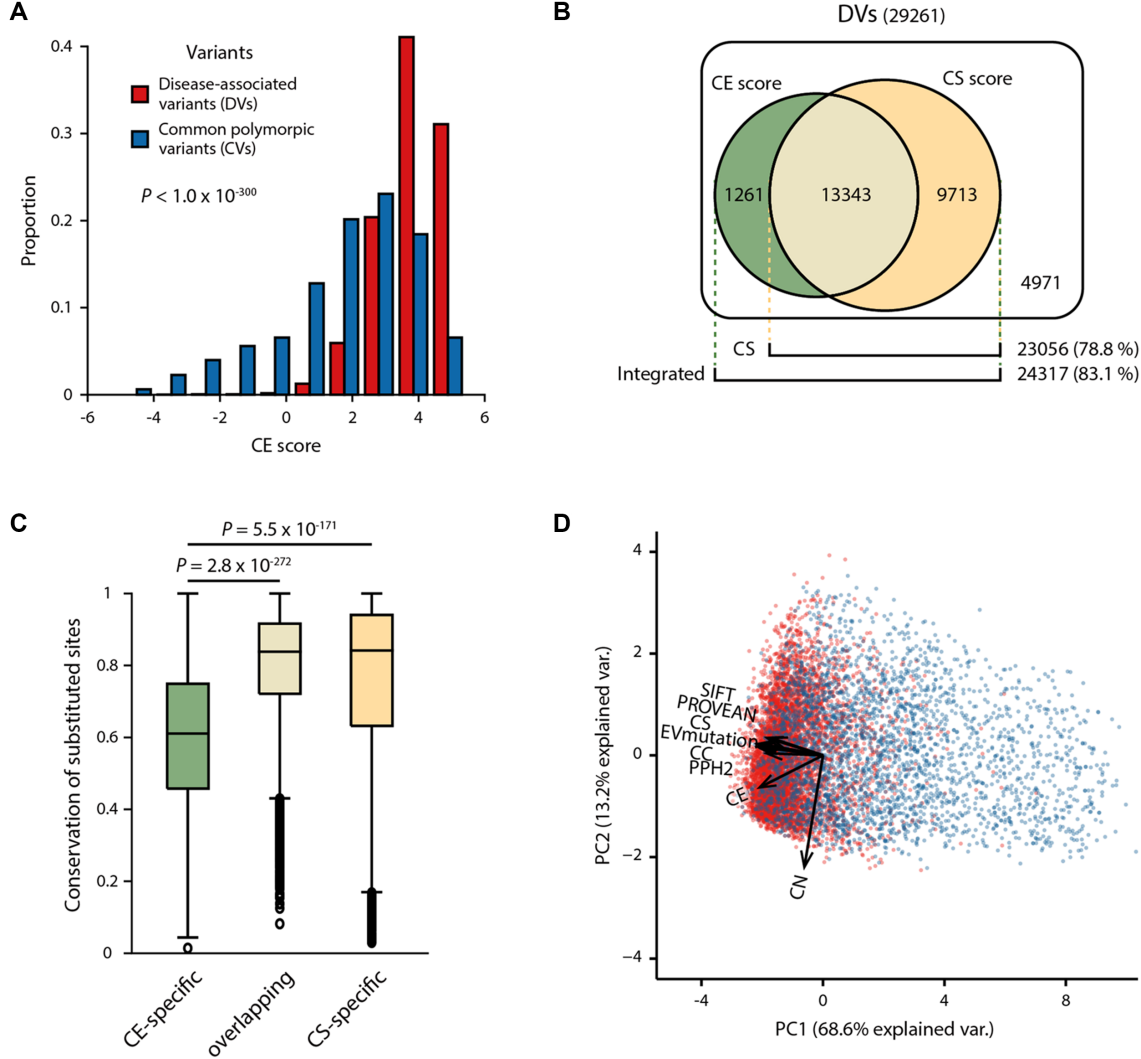
Prediction of DVs and CVs using CE and conservation-based scores. (**A**) Distribution of CE scores for DVs (red) and CVs (blue). (**B**) Venn diagram presenting the number of DVs correctly predicted by the CE (green) or CS (yellow) scores. (**C**) Conservation of sites with CE-specific, overlapping and CS-specific DVs. (**D**) PCA plot showing the eight scores from various methods (SIFT, PROVEAN, CS, EVmutation, CC, Polyphen2 [PPH2], CE and CN scores) projected onto the first two principal components. Dots correspond to the DVs (red) and CVs (blue) and vectors indicate the direction and strength of variants of each score.

We found that the CE score predicted human disease-associated variants at less-conserved sites by calculating the CE scores for 29 288 DVs and 39 167 CVs that were annotated in UniProt (37) (Supplementary Data S2). We found that the DVs had significantly higher CE scores than the CVs (Figure 4A; *P* < 1.0 × 10^−300^, Mann–Whitney U test). The average CE score for the DVs was 3.78, whereas that of the CVs was 1.82. This not only demonstrates that the CE score offers predictive power for identifying DVs but more generally confirms the validity of using the CE score to predict the variant impacts from a more diverse protein set (*n* = 12 277).

To determine the predictive ability of the CE score, we analyzed the ROC curve (Supplementary Figure S11). The AUC curve for the CE score was 0.82, which was comparable to the conservation-based scores of the same variants (CS, AUC = 0.83; SIFT, AUC = 0.81). Moreover, the DVs newly discovered by the CE score were found at less-conserved residues. We observed that 1261 DVs identified using the CE score were not identified by the CS score (Figure 4B). Indeed, CE-specific DVs were located at residues that were significantly less-conserved than the CS-specific DVs (Figure 4C; *P* = 5.5 × 10^−171^, Mann–Whitney U test). We found that 2203 DVs identified using the CE score, but not discoverable by SIFT scores, were indeed located at less-conserved residues (Supplementary Figures S12 and 13; *P* = 7.7 × 10^−61^, Mann–Whitney U test). Interestingly, 13 343 DVs identified from the overlapping predictions, the sites with the DVs predicted by both CE and CS scores, were more conserved than CE-specific DVs (Figure 4C; *P* = 2.8 × 10^−272^, Mann–Whitney U test). We further analyzed the CC and CN components of CE predictions and discovered that CE-specific predictions tended to have high CNs, whereas the overlapping prediction showed high CC contribution (Supplementary Figure S14).

### Integrating the CE score with conservation-based scores to identify more DVs

Because CE and conservation-based methods identified different DVs, we hypothesized that an integration of the CE score with other conservation-based scores would improve the prediction of DVs by taking more DVs (true positive) than CVs (false positive). To test the prediction performance, we applied Monte Carlo cross-validation and measured the various performance matrices. We found that the increase of sensitivity was larger than the loss of precision that occurred by integration (Supplementary Figure S15). Thus, the MCC, F1 score and balanced accuracy of the integrated scores were significantly higher than those of the conservation-based scores (Supplementary Figure S16). For example, the F1 score of the integrated (CE+CS) score was 0.78 ± 0.00, which was significantly higher than that of the CS score (0.77 ± 0.00; *P* = 9.6 × 10^−17^, Student's *t*-test). We found consistently improved performance when we used random forest classifiers to combine the CE and other conservation-based scores (Supplementary Figure S17). Furthermore, we validated the improvement of prediction using different sets of DVs and CVs from ClinVar and ExAC databases and found that the performance improvements were comparable to the predictions with the datasets taken from humsavar (Supplementary Figure S18).

We also explored the possibility that changing the threshold for the CS score would achieve a similar result, though it might also sacrifice some precision. However, we found that changing the threshold for the CS score resulted in a significant decrease in precision compared to that when integrating the CE and the CS scores (Supplementary Figure S19; *P* = 1.4 × 10^−64^, Mann–Whitney U test). These findings suggest that the integrated score can efficiently identify more DVs than relaxing the threshold used for conservation-based scores.

Together with significant improvement by the integration with CE, we hypothesized that CE is likely an orthogonal metric of conservation-based ones. Thus, we further analyzed the difference between the CE and conventional scores using PCA and found that the CE score has distinct explanatory power compared with that of other scores. Briefly, we performed PCA using eight scores (CN, CC, CE, CS, SIFT, Polyphen2, PROVEAN and EVmutation scores) and found that the vector of the CE score on PCA has a different direction than those of the conventional methods (Figure 4D). This finding suggests that the integration of the CE score with the conventional scores improves the predictive performance for DVs. At the same time, we also found that the vector of CN contributes less to the separation of DVs from CVs, which suggests that the combination of CN and CC scores into the CE score is necessary to predict variant impacts.

Furthermore, we found that the prediction performances of the integrated scores are better than the performances of other methods relying on coevolutionary analysis, such as EVmutation (17) and DeepSequence (52). Specifically, we compared the prediction performances of these methods by using Monte Carlo cross-validation based on repeats of random subsampling (90% for the training set and 10% for the test set). We found that the performances of our method were greater than EVmutation for predicting variant impacts of mutagenesis and human disease-associated residues (Supplementary Figures S20 and 21). Specifically, the F1 score, MCC and balanced accuracy of the integrated score (CE+CS) were significantly higher than those of EVmutation (*P* = 7.5 × 10^−20^ to 2.2 × 10^−249^, Student's *t*-test). In addition, we observed that our method performs comparable to DeepSequence for the prediction of variant impacts of mutagenesis (Supplementary Figure S20). Furthermore, one might ask that different approaches used in generating MSAs might contribute to the performance differences in identifying DVs. We confirmed that this is not the case. We found that CE scores calculated by different MSA files used in EVmutation and our method are highly correlated (Supplementary Figure S22).

We found that the integrated score identified DVs at sites that were less conserved, and these were not identified by current conservation-based methods. The ability to predict DVs at less-conserved sites was improved by the integrated approach, unlike using the CS score alone (Supplementary Figure S23). As an example, K329E of ACADM is a newly identified DV with a high CE score of 3.16. This variant is associated with MCAD deficiency (9). However, the conservation-based scores obtained using CS and SIFT did not identify the variant, because it is located at a site that is less conserved among homologous protein sequences.

We then asked which diseases were associated with the newly identified DVs by using the CE scores. We found that the newly identified DVs were associated with various disease classes (Table 2). To investigate these disease associations, we mapped 21 151 DVs to 21 disease classes on the basis of the physiological system affected (53). When we compared the use of integrated scores with the use of CS scores, we found that the coverage of identified DVs increased by an average of 4.3% for each disease class. For example, the CS score identified 4346/5124 DVs (84.82%) associated with metabolic diseases, whereas the integrated score (CE+CS) identified 4516 DVs (88.13%) that were associated with the same diseases. This result suggests that the integrated approach improved the detection of DVs that are associated with various diseases.

**Table 2.** The number and the ratio of identified DVs, determined using the CS and integrated scores across disease classes

		Identified DVs	
Disease class	whole DVs	CS	Integrated (CS + CE)
Metabolic	5124	4346 (84.82%)	4516 (88.13%)
Neurological	2642	1965 (74.38%)	2193 (83.01%)
Hematological	1393	1142 (81.98%)	1212 (87.01%)
Ophthalmological	1254	979 (78.07%)	1027 (81.90%)
Cancer	1121	819 (73.06%)	889 (79.30%)
Cardiovascular	1191	839 (70.45%)	871 (73.13%)
Endocrine	1011	779 (77.05%)	847 (83.78%)
Renal	970	775 (79.90%)	812 (83.71%)
Dermatological	830	667 (80.36%)	706 (85.06%)
Muscular	842	671 (79.69%)	704 (83.61%)
Connective tissue	635	564 (88.82%)	567 (89.29%)
Bone	562	503 (89.5%)	514 (91.46%)
Immunological	536	396 (73.88%)	426 (79.48%)
Skeletal	451	392 (86.92%)	411 (91.13%)
Developmental	469	374 (79.74%)	390 (83.16%)
Ear, Nose, Throat	295	245 (83.05%)	256 (86.78%)
Gastrointestinal	195	147 (75.38%)	154 (78.97%)
Respiratory	156	119 (76.28%)	131 (83.97%)
Nutritional	32	20 (62.50%)	20 (62.50%)
Psychiatric	15	8 (53.33%)	9 (60.00%)

The list of disease classes was sorted according to the number of DVs identified by the integrated (CE + CS) score.

### DVs identified by CE scores are enriched in protein–protein interaction interfaces

PPIs facilitate cellular functions; thus, the loss of PPIs affects phenotypic outcomes (54). Several human disease studies have shown that DVs tend to localize to PPI interfaces (55–57). However, residues at PPI interfaces are less likely to be conserved than residues in the protein's core; therefore, many DVs located in PPI interfaces are not discoverable with conservation-based methods. (55).

**Figure 5.**
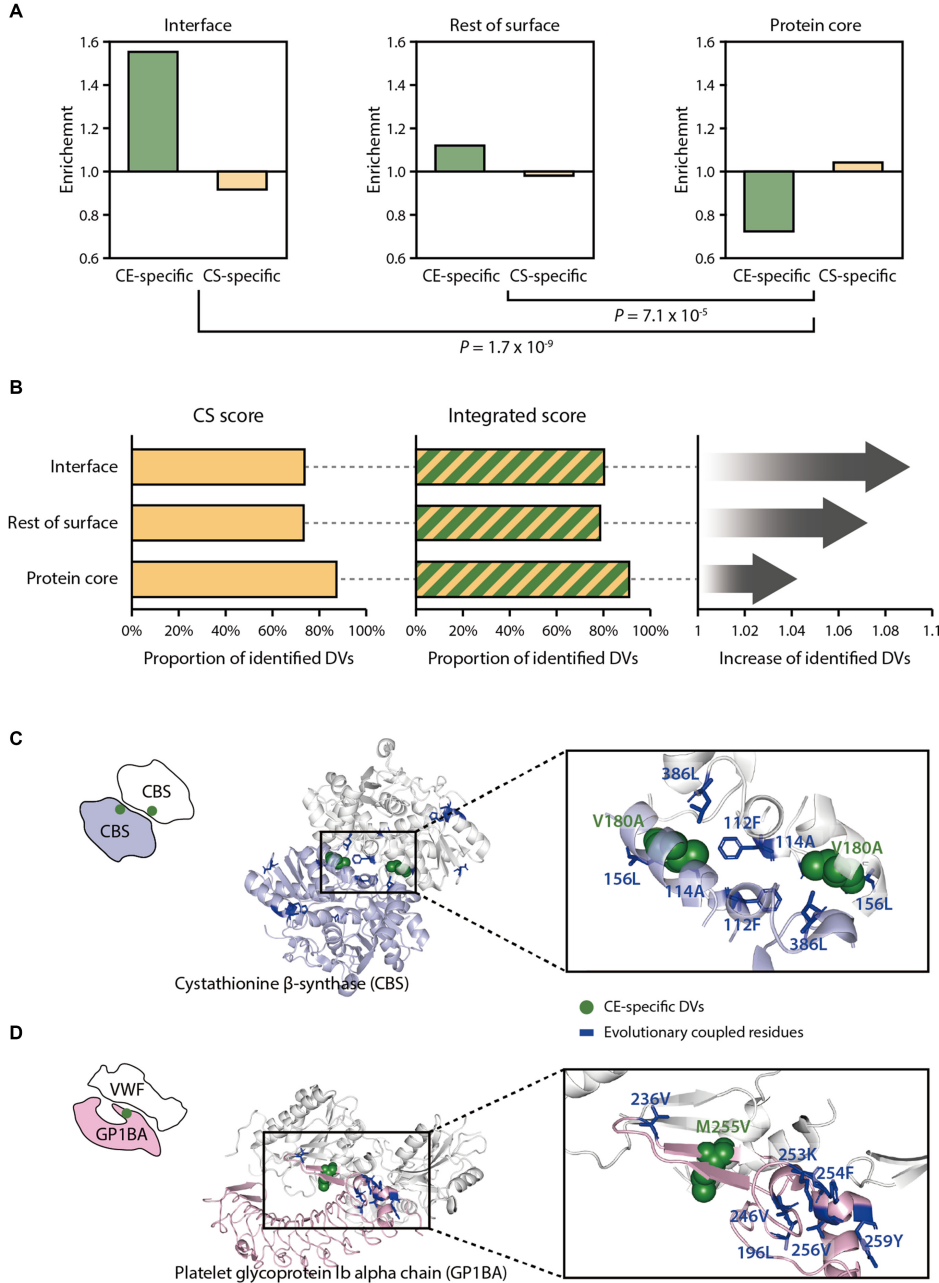
Structural characteristics of CE- and CS-specific DVs. (**A**) Enrichment of the CE- or CS-specific DVs at PPI interfaces, the rest of surface or protein core. Residues were classified as ‘interface’ using the known interfacial sites annotated in the Interactome INSIDER (42). Residues were classified as ‘the rest of surface’ or ‘protein core’ according to the relative solvent-accessible area, using a cutoff of 10%. (**B**) The left and the middle panels display the proportion of the identified DVs present on interface, the rest of surface or protein core regions when using the CS or the integrated score. The panel on the right shows the fold increase in the proportion of the identified DVs by the integrated score relative to the CS score. Examples of CE-specific DVs located on PPI interfaces of cystathionine β-synthase (PDB ID: 1JBQ) (**C**) and platelet glycoprotein 1b alpha chain (PDB ID: 1U0N) (**D**).

We found that using the CE score improved the identification of DVs in PPI interfaces. We analyzed the conservation of residues at the PPI interface and found that the PPI interface-located residues with DVs were less conserved than residues in the protein core (Supplementary Figure S24; *P* = 3.3 × 10^−34^, Mann–Whitney U test). Given that use of the CE score improved DV predictions at less-conserved residues, we expected the integrated approach would identify more DVs located in PPI interfaces. To analyze the structural features of the identified DVs, we investigated 179 528 PPI interface residues in 4150 PPIs using Interactome INSIDER (42). We also analyzed protein structures from PDB to classify noninterface residues as protein core residues and the rest of the surface residues. We found that CE-specific DVs were enriched at PPI interfaces, whereas the CS-specific DVs were enriched in the protein core (Figure 5A; *P* = 1.7 × 10^−9^, Fisher's exact test). Thus, the integrated score identified more DVs in PPI interfaces than the CS score alone (Figure 5B). The integrated score identified 80.3% of the DVs in the interfaces, whereas the CS score identified only 73.8% of them. Furthermore, we discovered that the DVs detected by CE scores tended to have significantly higher solvent accessibility than those detected by CS or SIFT scores (Supplementary Figure S25; *P* = 2.0 × 10^−8^ to 1.2 × 10^−12^, Mann–Whitney U test). This suggests that CE-specific DVs are located at the protein surface.

We identified a DV found at a PPI interface, V180A of cystathionine β-synthase (CBS), which is associated with homocystinuria (MIM: 236200). This disease causes a variety of clinical phenotypes, including mental retardation, lens dislocation and skeletal abnormalities. This variant was not identified by a conservation-based method (SIFT score = 0.17). However, the CE score identified this variant (CE score = 3.28), and we also found that the V180 residue is located at the PPI interface of a CBS homotetramer (Figure 5C). Importantly, the V180A variant is reported to reduce homotetramer formation and decrease CBS activity (58). When we closely examined the location of the evolutionarily coupled residues of the variant, we found that many evolutionarily coupled residues were located near the interfacial region, suggesting that correlated mutations tend to occur within the vicinity of the variant sites (59).

As another example of a CE-specific DV identification, we identified the M255V variant of the platelet glycoprotein Ib alpha chain (GP1BA), which is associated with pseudo-von Willebrand disease (VWDP). This disease is known to cause intermittent thrombocytopenia and a prolonged bleeding time (60). This variant was not identified by the conservation-based method (SIFT score = 0.11) but was identified by the CE score (CE score = 3.39). The M255 residue is located at the PPI interface of GP1BA and von Willebrand factor (Figure 5D). When we examined the surrounding residues of the DV positions, we found that many evolutionarily coupled residues were located near the variant position proximal to the interfacial region. Notably, the variant is reported to have an increased affinity for the interacting partner protein (61).

Furthermore, we investigated the enrichment of CE-specific DVs at various interface types, such as ligand, peptide, ion, DNA, and RNA interfaces. The CE-specific DVs tended to locate at the protein–ligand interfaces compared to CS-specific DVs (Supplementary Table S1; *P* = 0.02, Fisher's exact test). Specifically, 25.9% of CE-specific DVs were found at protein–ligand interface residues, whereas 21.3% of CS-specific DVs were found at protein-ligand interface regions. However, we did not find significant enrichment of CE-specific DVs at peptide, ion, DNA and RNA binding regions, potentially due to the limited coverage of the database.

## DISCUSSION

DVs located at less-conserved sites are difficult to identify in GWASs (8,62), because most GWASs rely on conservation-based methods to differentiate between DVs and neutral variants. However, some functional residues are not conserved across species. Additionally, the environmental conditions encountered by different species may require an alteration of protein function (63). Therefore, residues that regulate protein function, such as allosteric sites or PPI interfaces, may be less conserved in homologous proteins (64–66). In this study, we developed a scoring method called the CE score, which is based on the evolutionary coupling number of variant sites (Figure 1). We discovered that the CE score identified DVs at less-conserved residues that could not be identified by conservation-based methods (Figures 3B and 4C).

Initially, the CE score based on an evolutionary coupling number appears similar to a method based on evolutionary coupling strength, such as EVmutation (17). However, we found that the CE score and the EVmutation somehow identified different DVs (Figure 4D). This result is consistent with the previous report that the coupling strength and the coupling number identified different residues in a protein (30). Specifically, DVs predicted by our method are more likely located at less-conserved sites than those predicted by EVmutation (Supplementary Figure S26). Our method counts how many evolutionary couplings a variant site has. Thus, the sites with many covarying residues tend to be less conserved, because covariation is a result of compensatory amino acid changes. These two models are based on different premises, and they may be applicable for different purposes (67).

We found that the CE-specific DVs that were not identified by conservation-based methods were enriched at PPI interfaces (Figure 5A). Several lines of evidence suggest that interfacial residues are less conserved than protein core residues, because species often rewire their interactome over the course of evolution (66,68–69). Because of these changes in the interactome, the conservation-based methods perform poorly in identifying the DVs located at PPI interfaces (Figure 5B). However, we found that the CE score improved DV detection at PPI interfaces.

It is possible that residue variants at PPI interfaces perturb PPIs and cause disease (70), highlighting the importance of detecting DVs at protein interfaces. Several methods to identify DVs at protein interfaces have been developed (71–73), but those methods generally rely on structural information. Thus, the potential for DV detection has been limited by the availability of protein structures. We expect that use of the CE score will help facilitate the identification of additional DVs at PPI interfaces by using protein sequence information.

We found that the ability of CE scores to predict DVs depends on the quality of sequence samplings. Specifically, variant protein sequences with very narrow or very wide ranges of relatedness among homologous proteins were not well predicted by the CE score (Supplementary Figure S27). The reliability of evolutionary information depends on sequence sampling within the MSAs (26). Highly variable homologous sequences may provide inaccurate sequence alignments, resulting in background noise in MSAs that lead to misguided evolutionary analysis; by contrast, highly conserved homologous sequences mask or minimize the coupling signals (74,75). To address this problem, several studies have attempted to correct and optimize biased sequence sampling within MSAs (75,76).

Consensus approaches to the CE score and other conventional methods have considerable potential to improve the prediction of variant impacts. Our results indicate that integrating the CE score with conservation-based scores, CS and SIFT scores, improves the ability to detect DVs (Supplementary Figures S16–S18). Other consensus tools were also developed for predicting the impacts of variants by integrating various methods (77,78) to improve predictive accuracy. In our results, we showed that the CE score covered different DVs than other conventional conservation-based methods (Figure 4B). Thus, we anticipate that our method can complement conventional methods and be potentially useful when used with consensus approaches to improve the prediction of the impacts of DVs.

## DATA AVAILABILITY

Many extensions of our method are possible, including the attempts to predict the impact of human genetic variants. We provide precalculated CE scores for all possible human gene variants at https://sbi.postech.ac.kr/w/CE.

## Supplementary Material

gkz536_Supplemental_Files
