# Supplementary material for: Evolutionary coupling analysis identifies the impact of disease-associated variants at less-conserved sites
Source: Nucleic Acids Res. 2019 Jun 14;47(16):e94. doi: 10.1093/nar/gkz536 (PMC6895274; doi:10.1093/nar/gkz536)
Supplement: gkz536_Supplemental_Files [file gkz536_supplemental_files.zip › final_revised_Kim_CEscore_Supple.pdf]

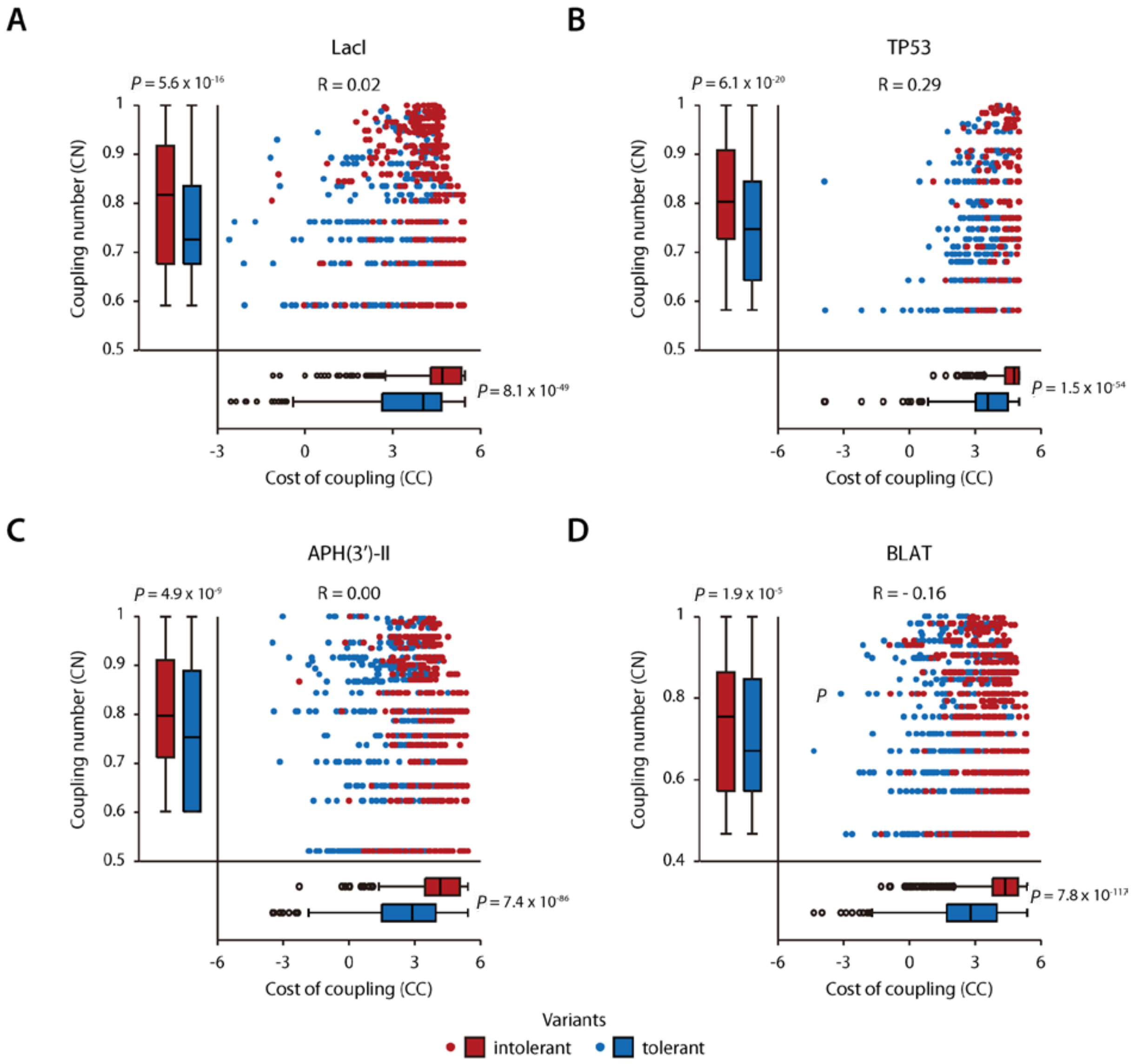

Supplementary Figure S1. CN and CC distributions of intolerant (red) and tolerant (blue) variants from LacI, TP53, APH(3′)-II, and BLAT.

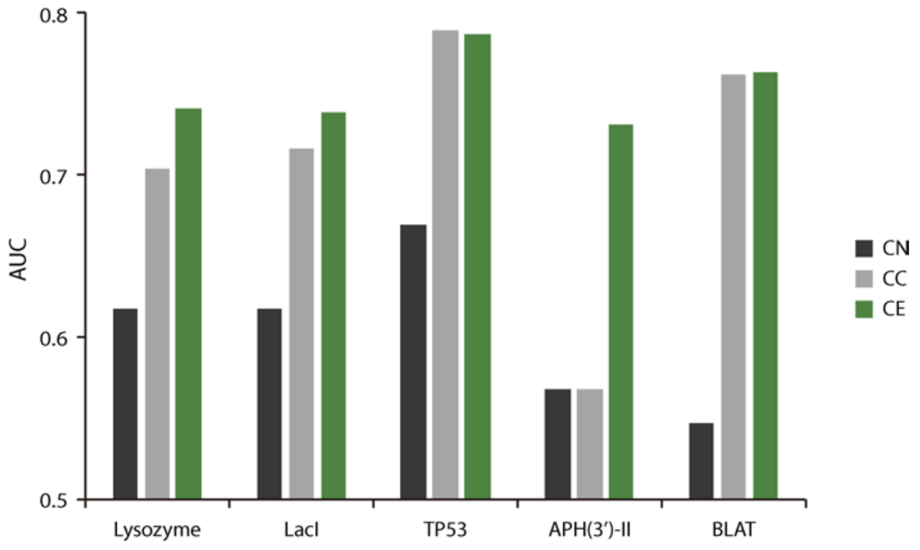

Supplementary Figure S2. AUCs of the CN (black), CC (gray), and CE (green) scores for predicting the impacts of variants from mutagenesis studies.

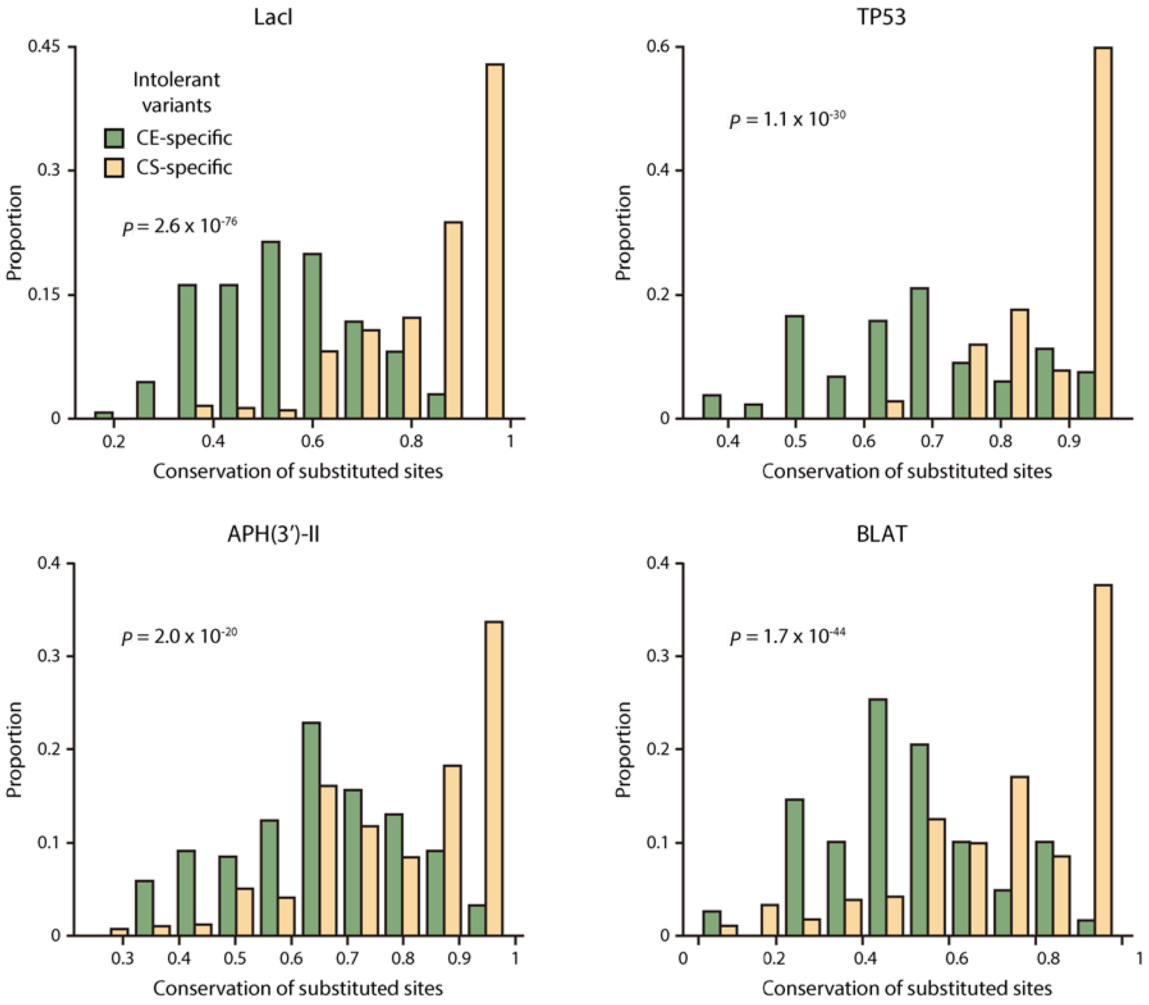

Supplementary Figure S3. Conservation of CE-specific (green) or CS-specific (yellow) intolerant variants of LacI, TP53, APH(3′)-II, and BLAT.

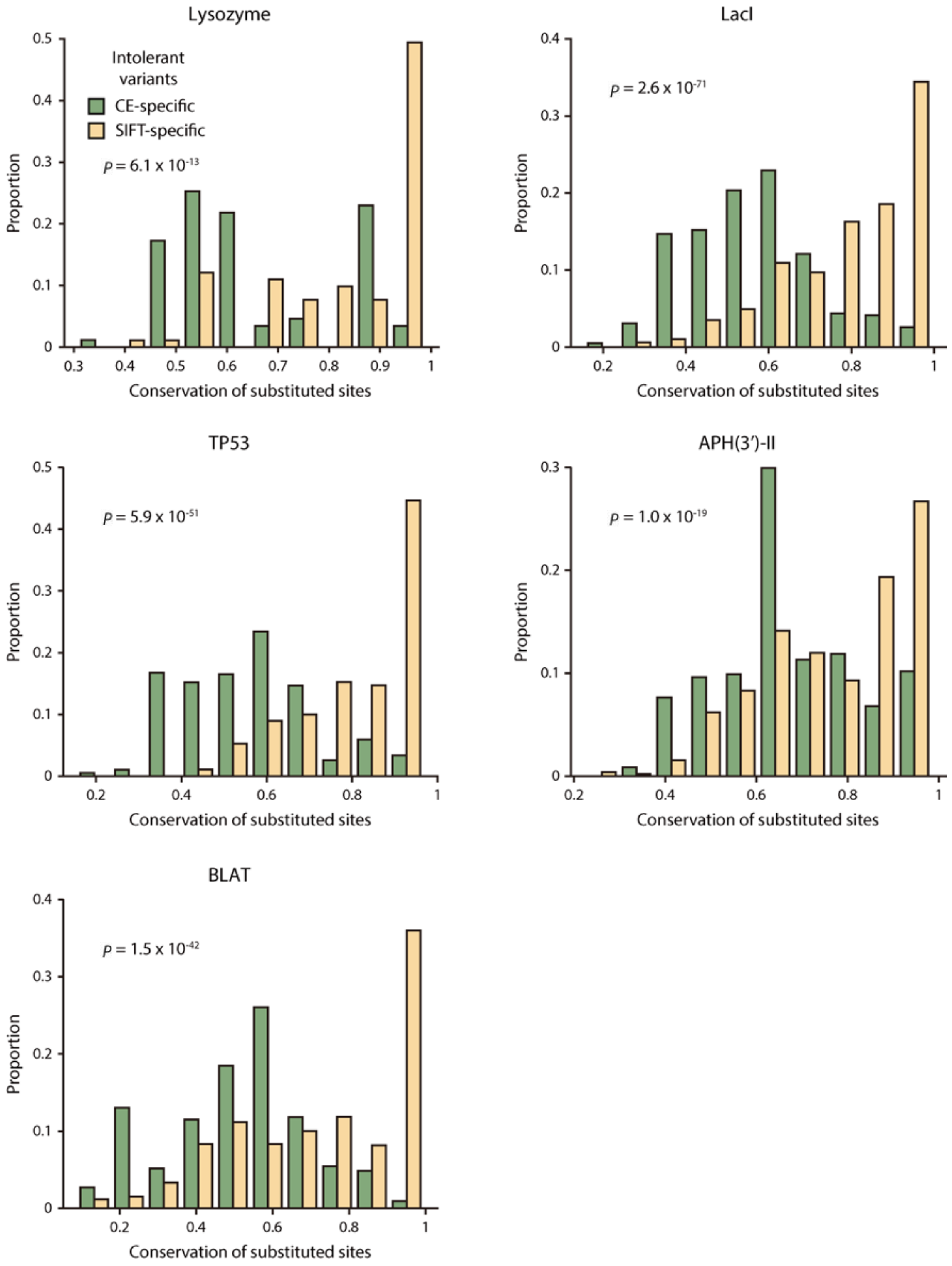

Supplementary figure S4. Conservation of CE-specific (green) or SIFT-specific (yellow) intolerant variants of LacI, TP53, APH(3′)-II, and BLAT.

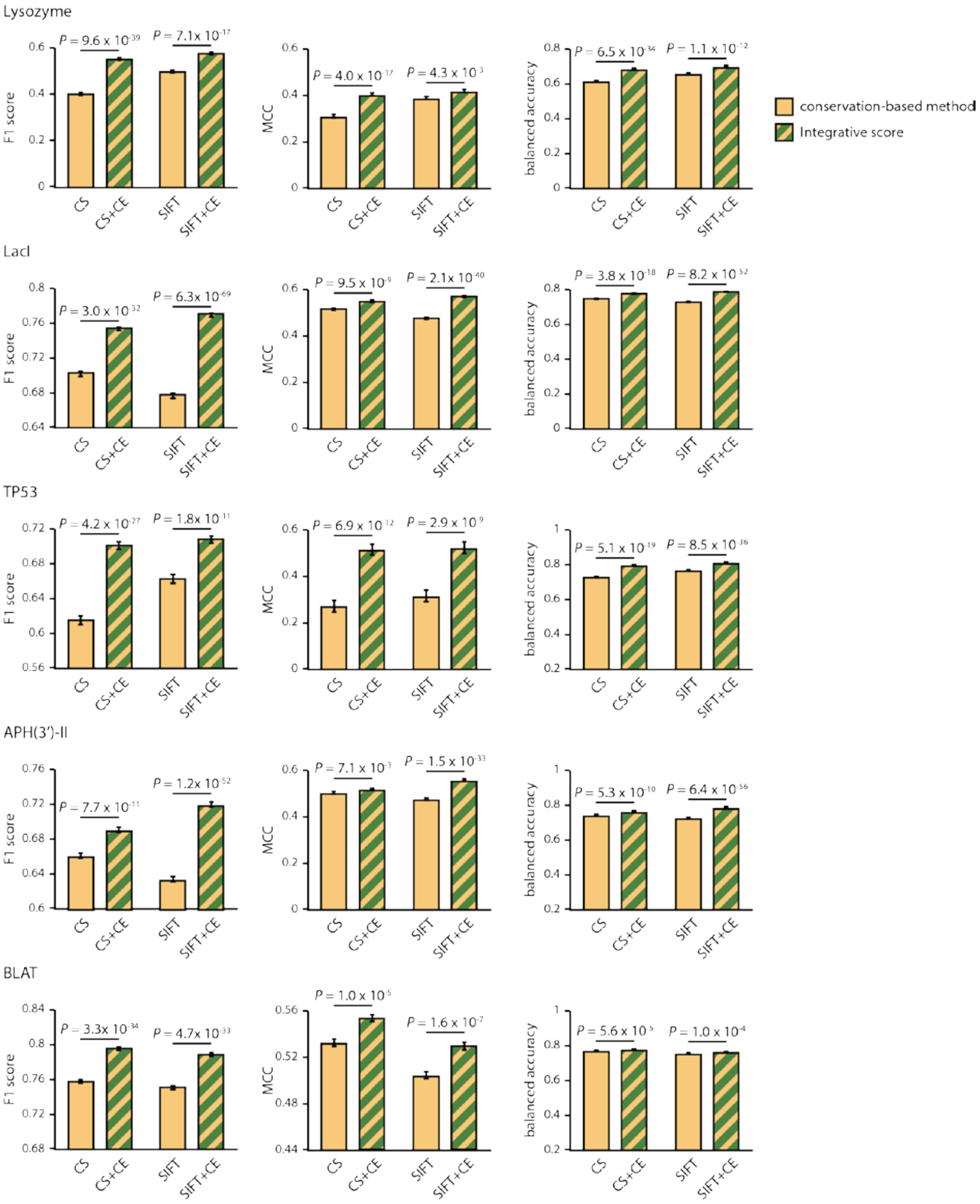

Supplementary Figure S5. Prediction performances of assessing the impacts of variants from mutagenesis studies by using the CS (yellow) and the integrated (CE+CS; yellow and green stripes) scores.

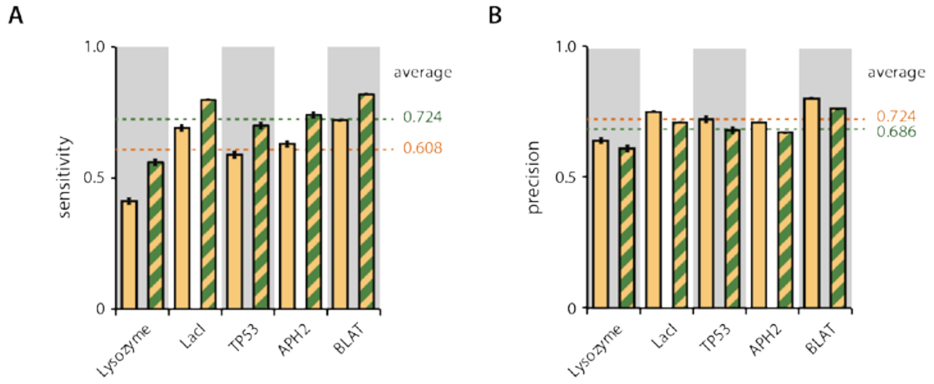

Supplementary Figure S6. Sensitivities and precisions for the CS (yellow) and the integrated (CE+CS; yellow and green stripes) scores for the variants from mutagenesis studies.

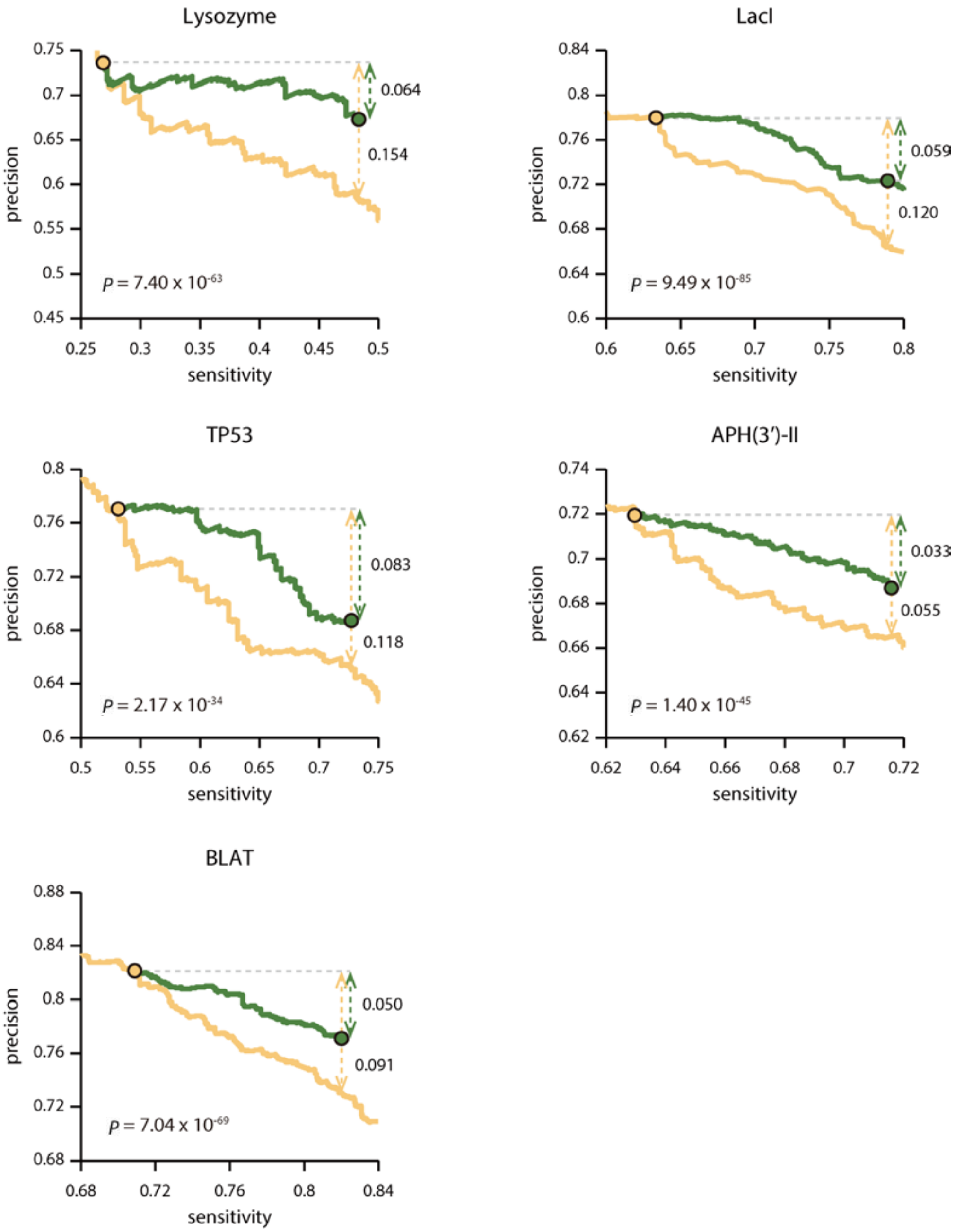

Supplementary Figure S7. Precision while lowering the thresholds until the sensitivity of CS (yellow) score is equal to the sensitivity of integrated (CE+CS; green) scores to predict the impacts of variants from mutagenesis studies.

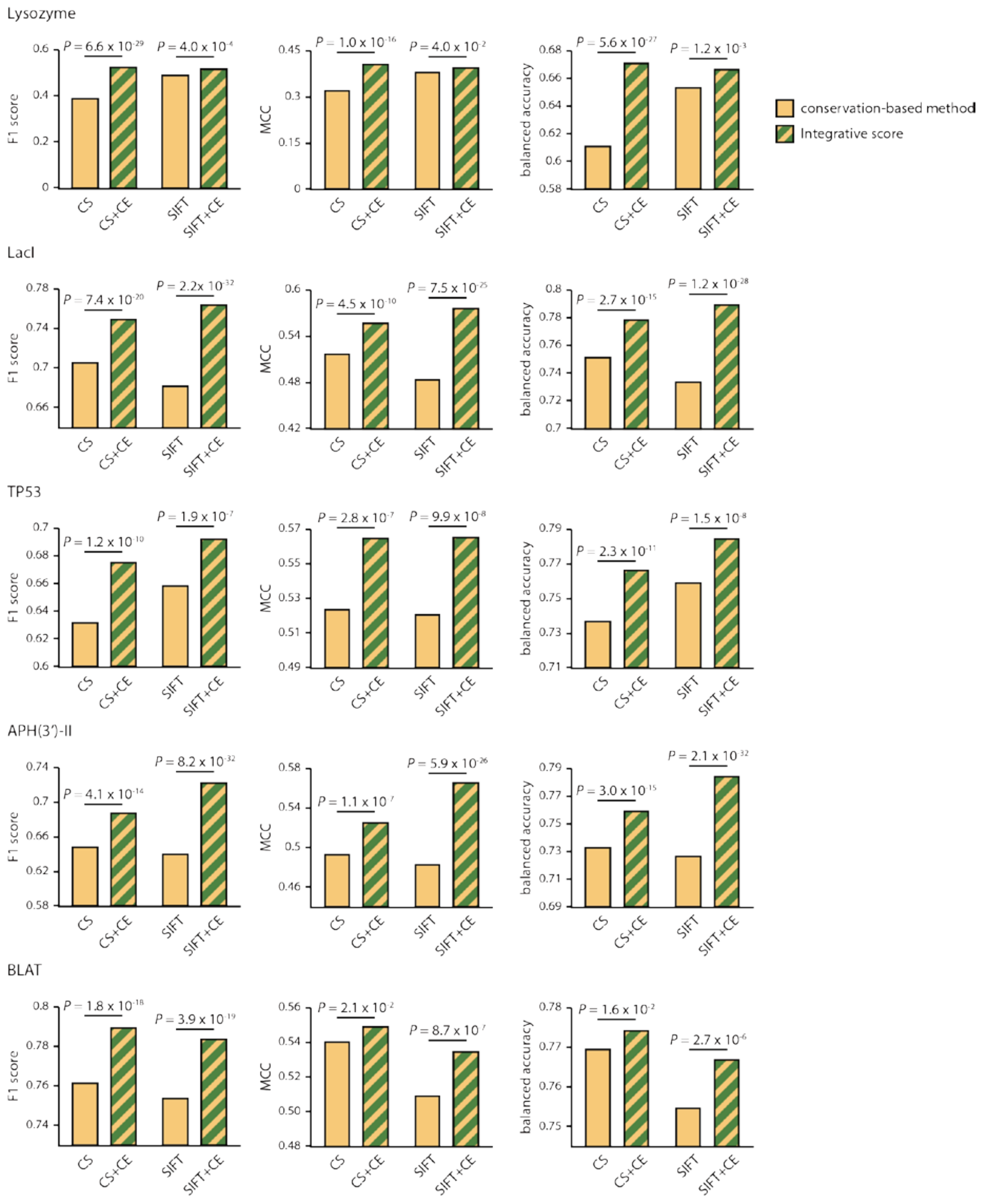

Supplementary Figure S8. Prediction performances of the random forest classifiers for assessing the impacts of variants from mutagenesis studies by using the CS (yellow) and the integrated (CE+CS; yellow and green stripes) scores.

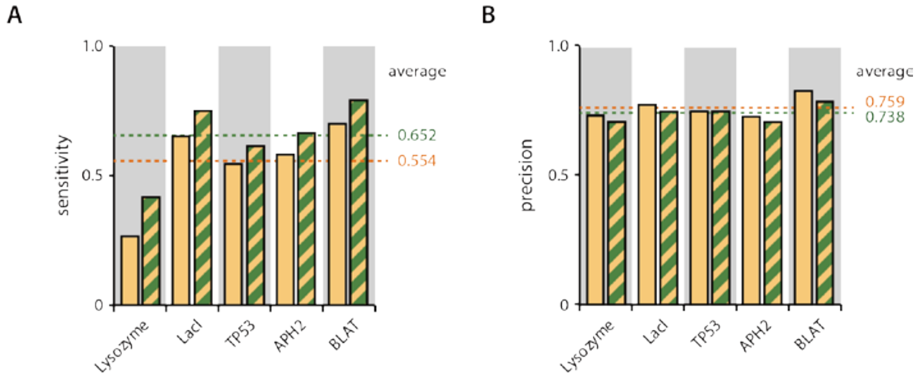

Supplementary Figure S9. Sensitivities and precisions of the random forest classifiers for assessing the impacts of variants from mutagenesis studies with CS (yellow) score and those with integrated (CE+CS; yellow and green stripes) scores.

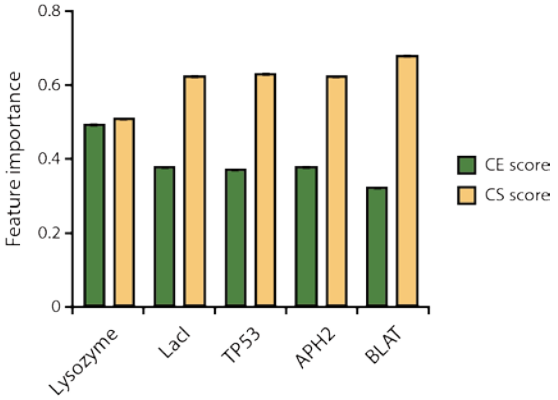

Supplementary Figure S10. Average feature importance of the CE and CS scores estimated by random forest classifiers with Monte Carlo cross-validation (100 times).

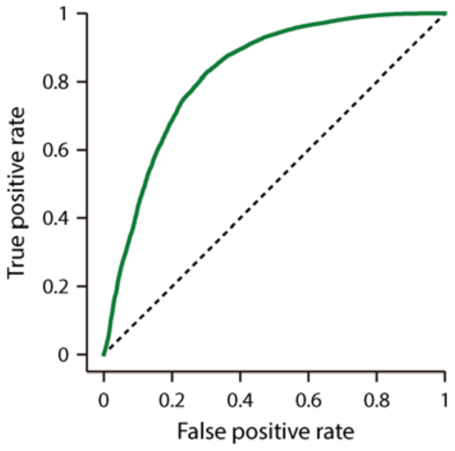

Supplementary Figure S11. The ROC curve for the CE score to predict DVs and CVs.

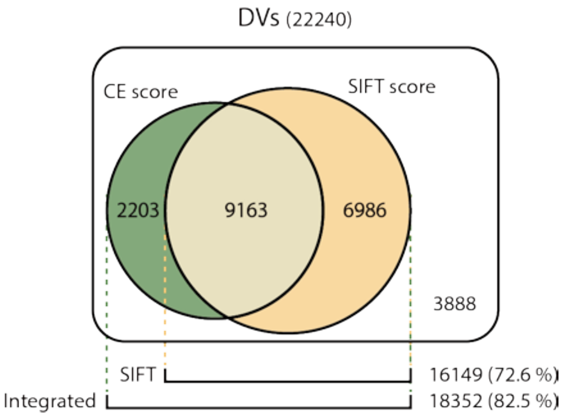

Supplementary Figure S12. Venn diagram presenting the number of DVs correctly predicted by the CE (green) or SIFT (yellow) scores. The total number of DVs indicates those that were identified by at least one of the CE or SIFT scores.

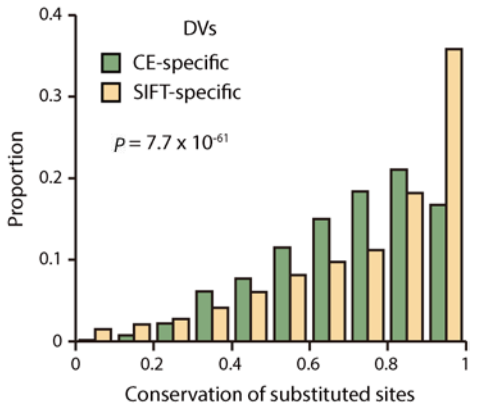

Supplementary Figure S13. Conservation of CE-specific (green) or SIFT-specific (yellow) DVs.

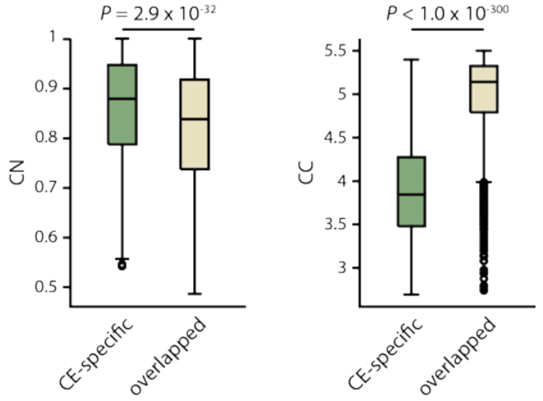

Supplementary Figure S14. CN and CC scores of the CE-specific or overlapped DVs.

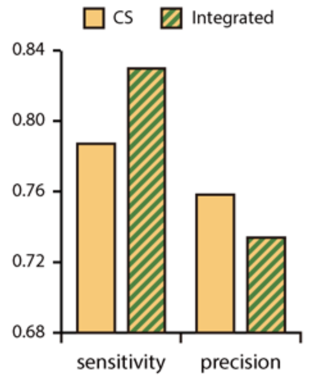

Supplementary Figure S15. Sensitivity and precision of the CS and the integrated (CE+CS) scores for predicting DVs and CVs.

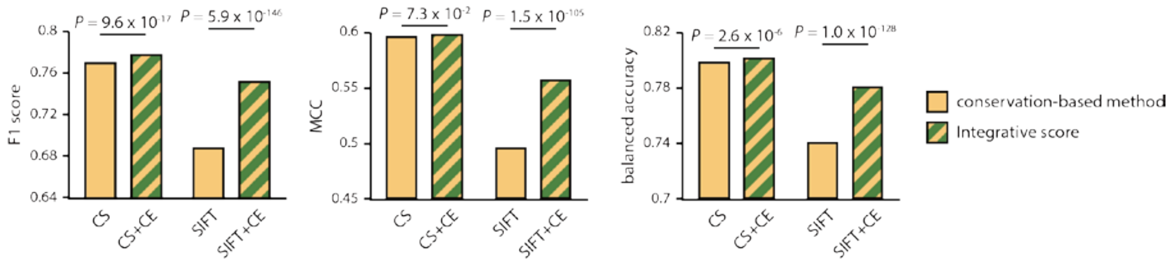

Supplementary Figure S16. Prediction performances for assessing the impacts of variants by using the CS (yellow) and the integrative (CE+CS; yellow and green stripes) scores for DVs and CVs annotated in humsavar.

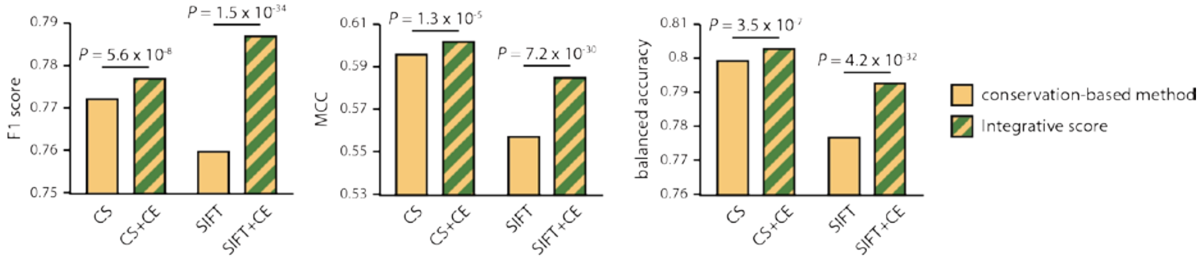

Supplementary Figure S17. Prediction performances of random forest classifiers for assessing the impacts of variants by using the CS (yellow) and the integrative (CE+CS; yellow and green stripes) scores for DVs and CVs annotated in humsavar.

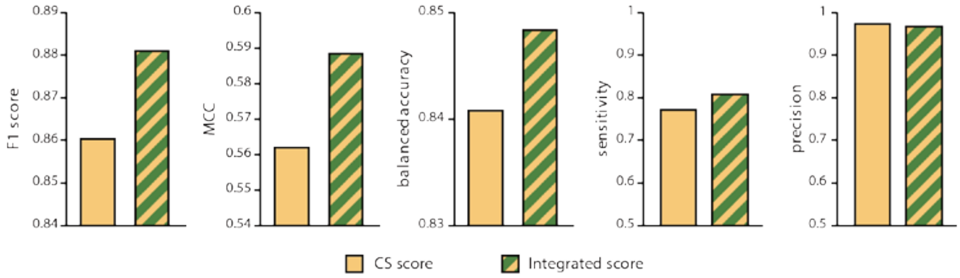

Supplementary Figure S18. Prediction performances for assessing the impacts of variants by using the CS (yellow) and the integrative (CE+CS; yellow and green stripes) scores for DVs and CVs annotated in ClinVar and ExAC, respectively. The thresholds were obtained by DVs and CVs annotated in an independent dataset, humsavar.

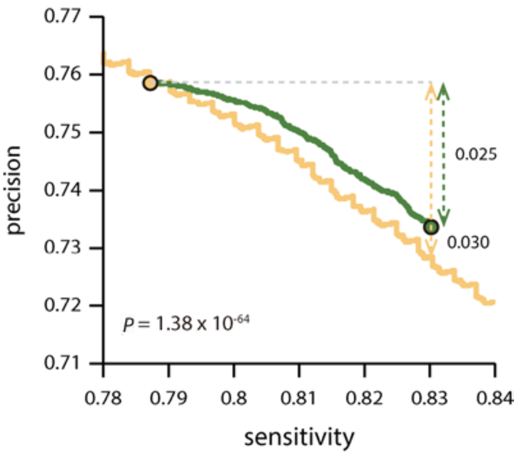

Supplementary Figure S19. Precision while lowering the thresholds until the sensitivity of CS (yellow) scores is equal to the sensitivity of integrated scores (CE+CS) (green) to predict DVs and CVs.

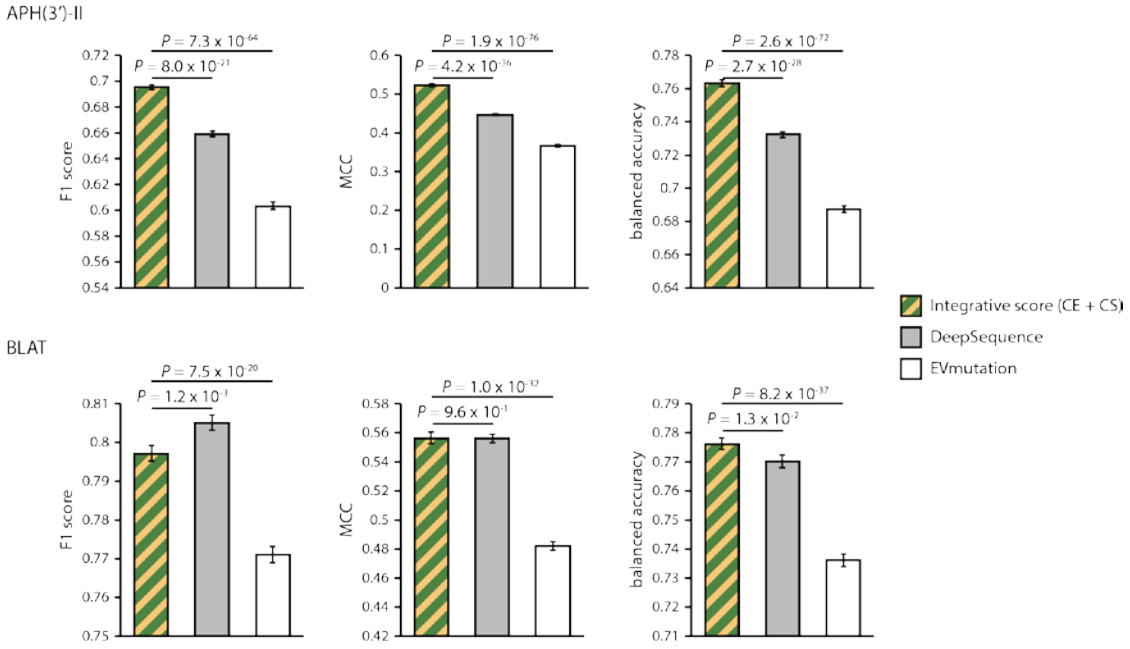

Supplementary Figure S20. Prediction performances for assessing the impacts of variants by using the integrative (CE+CS; yellow and green striped), DeepSequence (gray), and EVmutation (white) scores for the variants from mutagenesis studies.

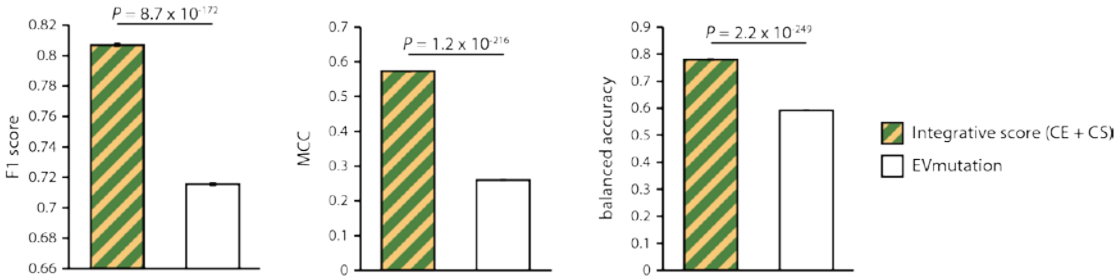

Supplementary Figure S21. Prediction performances for assessing the impacts of variants by using the integrative (CE+CS; yellow and green stripes) and EVmutation (white) scores for DVs and CVs.

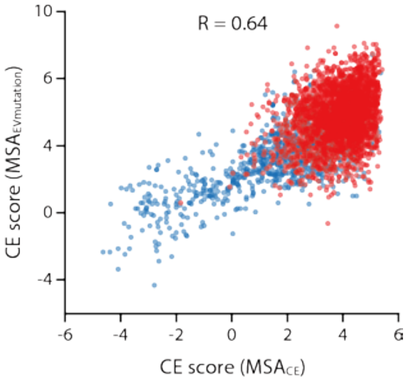

Supplementary Figure S22. The correlation between the CE scores calculated by the MSAs built by EVmutation and our analysis.

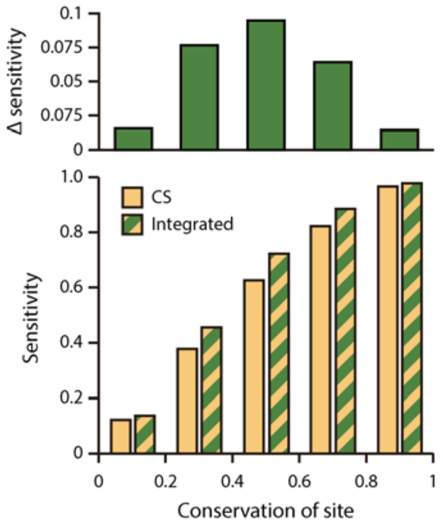

Supplementary Figure S23. The lower panel shows sensitivities of the CS and the integrated scores, according to the degree of conservation of DVs. The upper panel shows the difference in sensitivities between the CS and integrated scores.

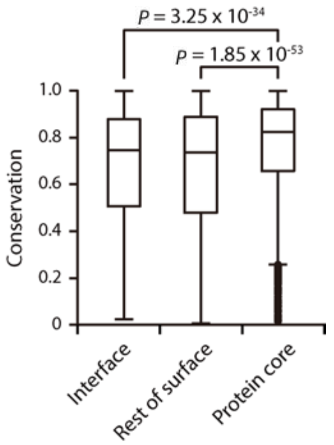

Supplementary Figure S24. Conservation of sites with DVs on the interface, the rest of surface, or in the protein core.

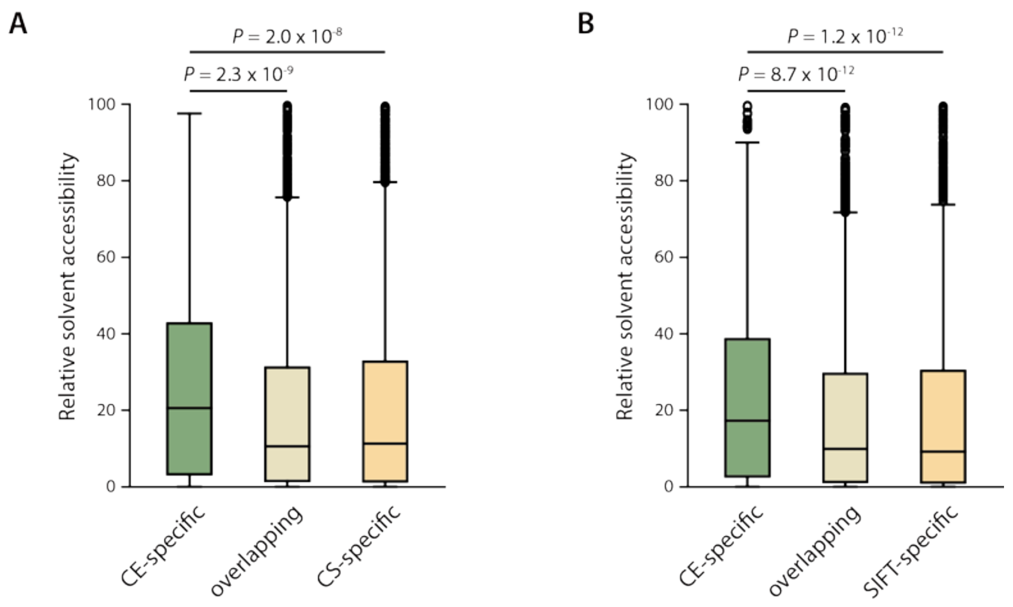

Supplementary Figure S25. The distributions of the relative solvent accessibility of the sites of DVs identified by the CE and conservation-based scores (CS or SIFT scores). The relative solvent accessibility was measured by NACCESS.

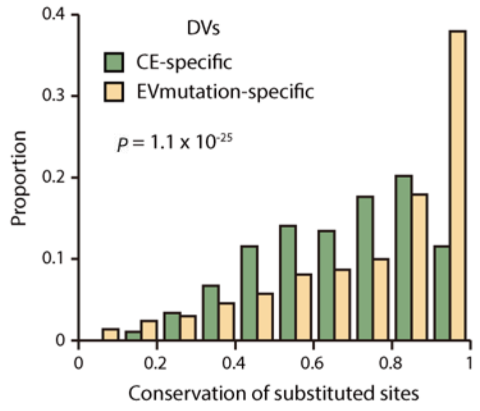

Supplementary Figure S26. Conservation of sites with CE-specific (green) or EVmutation-specific (yellow) DVs.

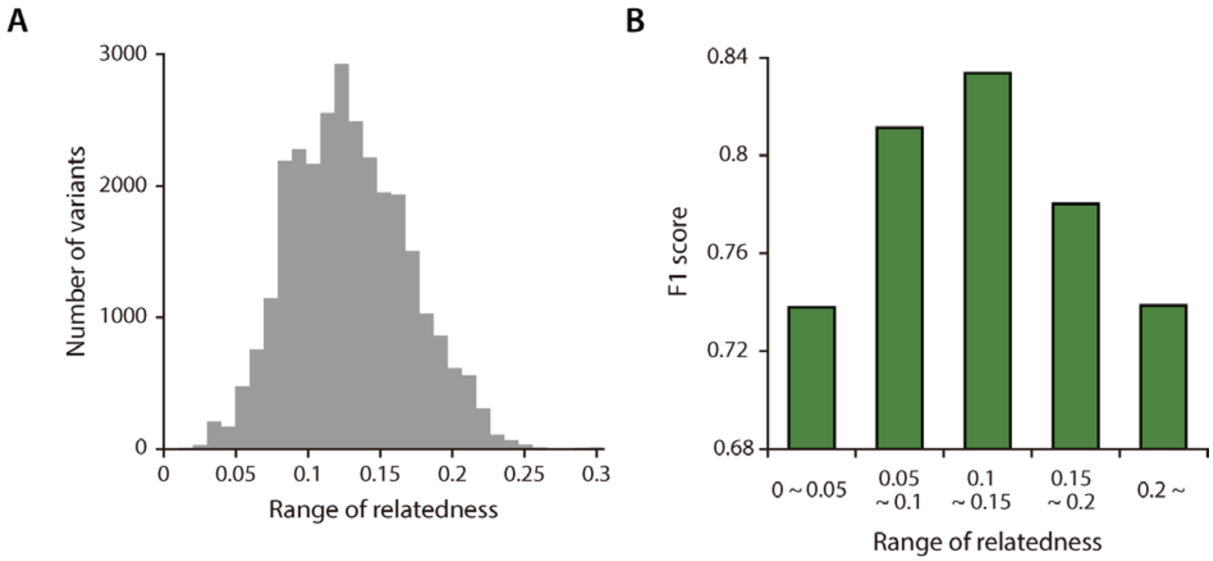

Supplementary Figure S27. Sequence relatedness of the examined proteins. (A) Distribution of relatedness range among homologous sequences. The range of relatedness was calculated using the standard deviation of sequence identity between a human protein with DVs and the homologous proteins in an MSA. (B) F1 score to predict DVs across five bins of relatedness ranges for examined proteins.

		DVs						P
		CE-specific		Overlapped		CS-specific		
PPI	Interface	227	**34.2%**	2,096	30.4%	1,361	27.1%	1.9×10^{-19}
	noninterface	437	65.8%	4,789	69.6%	3,652	**72.9%**	
LIG	Interface	148	**25.9%**	1,564	25.0%	969	21.3%	0.02
	noninterface	424	74.1%	4,694	75.0%	3,571	**78.7%**	
ION	Interface	16	3.3%	313	6.0%	193	5.1%	0.14
	noninterface	466	96.7%	4,940	94.0%	3,599	94.9%	
PEP	Interface	24	9.3%	280	12.9%	166	11.5%	0.34
	noninterface	234	90.7%	1,894	87.1%	1,279	88.5%	
DNA	Interface	7	10.0%	166	23.6%	74	16.4%	0.21
	noninterface	63	90.0%	536	76.4%	376	83.6%	
RNA	Interface	3	8.3%	29	7.6%	24	9.9%	1.00
	noninterface	33	91.7%	355	92.4%	219	90.1%	

Supplementary Table S1. Enrichment of the CE- or CS-specific DVs at protein-protein interaction (PPI), and protein-ligand (LIG), -ion (ION), -peptide (PEP), -DNA (DNA), and -RNA (RNA) interfaces. The interfacial residues annotations were taken from the IBIS.
